# Australian link worker social prescribing programs: An integrative review

**DOI:** 10.1371/journal.pone.0309783

**Published:** 2024-11-11

**Authors:** James R. Baker, Michelle Bissett, Rosanne Freak-Poli, Genevieve A. Dingle, Yvonne Zurynski, Thomas Astell-Burt, Eric Brymer, Tina Prassos, Tamsin Thomas, Cassandra Tognarini, Christina Aggar

**Affiliations:** 1 Faculty of Health, Southern Cross University, Gold Coast, Queensland, Australia; 2 Australian Social Prescribing Institute of Research and Education, Sydney, New South Wales, Australia; 3 Primary & Community Care Services, Sydney, New South Wales, Australia; 4 Department of Medicine, School of Clinical Sciences at Monash Health, Melbourne, Victoria, Australia; 5 School of Psychology, The University of Queensland, Brisbane, Queensland, Australia; 6 Australian Institute of Health Innovation, Macquarie University, Sydney, New South Wales, Australia; 7 School of Architecture, Design, and Planning, University of Sydney, Sydney, New South Wales, Australia; 8 Population Wellbeing and Environment Research Lab (PowerLab), Sydney, New South Wales, Australia; University of Georgia, UNITED STATES OF AMERICA

## Abstract

Link worker social prescribing programs are gaining recognition in Australia for addressing health and social needs outside routine medical care. The evaluation of these programs is essential for informing future social prescribing programs, research and evolving policy. However, diverse outcome evaluation measures present challenges for benchmarking across link worker social prescribing programs. An integrative review was conducted to identify and describe outcome domains and measures, and the methodological approaches and evaluation designs of link worker social prescribing programs in Australia. Comprehensive searches of the literature on link worker social prescribing programs in Australia were conducted across 14 electronic databases. In order to reduce the risk of bias, study selection and data extraction were conducted independently by multiple authors, and included studies underwent quality and risk of bias assessment using the standardised Mixed Methods Appraisal Tool. Six studies met the inclusion criteria. Outcome domains were categorised into ‘person-level’, ‘system-level’ and ‘program implementation’ domains. Despite the variation in participant groups, the ‘person-level’ domains of global well-being and social well-being were consistently evaluated. While measurement tools varied significantly, the WHO Quality of Life Brief Assessment and short-form UCLA Loneliness Scale were most commonly applied. At the system level, health service utilisation was primarily evaluated. This integrative review reports on the current state of evidence in Australia, with the potential to track changes and trends over time. Developing a core outcome set, incorporating stakeholder and consumer contributions for benchmarking aligned with the healthcare landscape is recommended. The findings may guide the refining of social prescribing initiatives and future research, ensuring methodological robustness and alignment with individual and community needs.

## Introduction

The prevalence of complex health and social needs are increasing demands on Australian primary healthcare services [1] and General Practice/practitioners (GPs) are rapidly reaching capacity [2]. There are also notable disparities in healthcare service access, particularly among vulnerable population groups [2–5]. Existing healthcare systems lack the capacity to comprehensively address the social determinants of health, including social participation, employment, education, neighbourhood conditions, and other social and economic factors [6], which influence health and well-being [7]. Thus, additional approaches are imperative to advance person-centred care and improve health outcomes [8].

Social prescribing offers a person-centred and scalable solution to unmet health and social needs, by leveraging community assets to facilitate collaboration across health, social and community services [8, 9]. This multidisciplinary approach addresses healthcare system constraints without depending solely on routine medical care. UK evaluations have consistently found positive social return on investment for social prescribing schemes [10, 11], with a similar outcome found in Australia [12]. However, these evaluations primarily assess costs to the health system and overlook expenses related to non-medical service utilisation. Ultimately, the person-centred and collaborative nature of social prescribing empowers individuals by equipping them with the necessary knowledge, skills, motivation, and confidence to manage their own health and social well-being, promoting sustained benefits [8].

Globally, social prescribing is implemented through two main models: the direct referral model and the link worker model. In the direct referral model, health professionals (e.g. GP, community worker, social worker) refer patients directly to non-medical community-based programs or activities to improve health and well-being [9]. The link worker model involves a referral to a link worker, who assists the person in developing and executing a personalised plan that utilises community-based resources to enhance their quality of life [13]. This model also focuses on building the individual’s confidence, skills and health literacy to manage their own unmet health and social needs, such as chronic conditions and loneliness [14]. Both models facilitate community engagement and reduce loneliness by promoting engagement in physical activity, arts, culture and the natural world, or promoting access to practical advice and information [15].

In Australia, both models of social prescribing operate, however the link worker model presents several advantages [16]. Firstly, link workers possess a higher level of knowledge of local services compared to GPs, and can dedicate additional time to further engage with clients and assess which services, programs, and activities would be most appropriate [14]. Secondly, link workers can oversee follow-up on health-related non-medical challenges without the cost of an additional GP appointment, with the potential for integrated GP feedback systems [17, 18]. Given Australia’s diverse demographics, where 27.6% of the population is born overseas [19] and 33.1% reside in regional or rural areas [20], the link worker model is vital. This model is better equipped to accommodate the diverse social determinants of health inherent in the population characteristics and address the wide spectrum of health and social care needs [8, 9].

Social prescribing has gained traction globally over the past decade [8], with a recent review identifying 159 social prescribing programs for adults internationally [21]. In Australia, there is no funding model or national framework, however the adoption of social prescribing is rapidly increasing [15]. This trend aligns with the Australian government’s ‘National Preventive Health Strategy 2021 to 2030’, which advocates for the integration of social prescribing to prevent public health concerns, and Australia’s 10 Year Primary Health Plan 2022–2032, which charges Primary Health Networks to trial models of social prescribing nationally. Currently, the Mitchell Institute is conducting a feasibility study commissioned by the Australian Department of Health and Aged Care to explore social prescribing within primary health care. While international experiences provide valuable insights, local factors, such as the remoteness of the Australian outback and regionality, culturally and linguistically diverse groups and differing funding systems across states and territories, necessitate a tailored approach to implementation within the Australian context.

Although international guidance exists for planning and implementing social prescribing programs [21], evaluating these programs remains a consistent challenge [15]. This is because social prescribing programs consist of many components and are complex, often consisting of individualised interventions that can range in type, purpose, number, duration and environment of delivery [1, 8]. There is a need for a ‘multi-criteria’ approach to effectively assess both the implementation process and the outcomes of social prescribing interventions [1]. However, challenges to this approach include data inconsistency, diverse measurement with limited comparability, difficulty in identifying influential aspects of the intervention, and inflexibility in the selection of evaluation strategies, all of which compromise data quality [1, 9, 22].

Consequently, to enhance the evaluation of social prescribing programs in the Australian context, a crucial initial step involves establishing standardized data collection protocols that are endorsed by stakeholders [22, 23]. This necessitates consensus on the outcome domains to be investigated and a directive for the adoption of validated outcome measures [23]. In this integrative review, we defined outcome domains as “broad, descriptive categories under which several, more specific outcomes might be grouped” [24] and outcome measures as “the tool used to assess the outcome” [25]. Establishing core outcome domains for key diagnostic groups and interventions is paramount as it allows for the aggregation and comparison of findings across studies, facilitating evidence-informed decision-making [26]. A core outcome set defines a standardised set of outcomes to be routinely measured and reported in intervention studies [24, 27, 28]. Despite the importance of core outcome sets, one has yet to be defined for social prescribing. Therefore, identifying and examining outcome domains and measures is an initial step to enable robust evaluation of social prescribing programs in Australia.

## Aims and objectives

This research aims to systematically explore the Australian social prescribing literature, focusing on the link worker model. We seek to chronicle the current Australian research, which will serve as a foundational reference to track social prescribing progression. By detailing ‘what’ has been measured and ’ how’, we aim to inform future research, guide evaluation practices, and influence policy decisions in the early stages of scaling up link worker social prescribing in Australia. Specifically, our objectives are to identify and describe:

1. outcome domains and outcome measures used to evaluate social prescribing programs in Australia;
2. methodological approaches and evaluation designs, lending insights into the foundational research methodologies employed.

## Method

An integrative review was conducted according to the methodology of Whittemore and Knafl [29] and includes both qualitative and quantitative studies in order to provide a comprehensive and nuanced understanding of current evidence. Studies were identified using academic and clinical trial database searches and reference list checking, and evaluated using the Mixed Methods Appraisal Tool [MMAT; 30].

### Search strategy

The search strategy was developed by two authors (JB, CA) in consultation with other experts in the field of social prescribing and an expert university librarian. A systematic and comprehensive search of the literature on link worker social prescribing programs in Australia was undertaken across 14 electronic databases in October 2023. These were: Academic Search Premier, Aushealth, APA PsycArticles, APA PsycINFO, CINAHL, Cochrane Library (including protocols and trials), Health Source: Nursing/Academic Edition, Humanities International Complete, Ovid (all journals), Psychology and Behavioral Sciences Collection, ProQuest (all databases), PubMed (includes Medline), Scopus, and Web of Science. An additional three clinical trial registries were searched: the Australian New Zealand Clinical Trials Registry, the WHO International Clinical Trial Registry Platform (ICTRP), and the Australian Cancer Trials Registry. Reference lists of included studies and related review papers were scanned, and experts in the field of social prescribing were consulted to identify additional studies.

Pilot testing of the search strategy was undertaken on 15 July 2023 on several databases, including PsycINFO, Proquest, and PubMed using a combination of relevant search terms including 1) "Social Prescri*"[All Fields]; 2) "Australia"[MeSH Terms] OR "Australia*"[All Fields]; 3) 1 AND 2; 4), and without a timeframe to increase the likelihood of identifying relevant published studies. The use of MeSH headings, search terms, wildcards, and limits were adapted to each database. Wildcards ensured all permutations of words were included. One search consisting of four steps conducted using MeSH headings, relevant terms and abbreviations was used: 1) "Social Prescri*"[All Fields]; 2) "Australia"[MeSH Terms] OR "Australia*"[All Fields]; 3) 1 AND 2; 4) limits to English language and journal articles. Following removal of duplicates, citation tracking and comprehensive inspection of reference lists were undertaken to identify additional relevant papers.

### Selection of sources of evidence

All database searches were conducted by author TT with results imported into Endnote X9 [31]. Inclusion and exclusion criteria are presented in Table 1. Initial double screening of identified records according to selection criteria was completed based on record titles and abstracts (conducted independently by authors TT, CT) and disagreement resolved by discussion. Full-text review of the remaining papers was conducted independently by four authors (CA, JB, TT, CT) and disagreement was resolved by discussion. Protocol papers were included except when the corresponding article with results could be accessed. In this scenario, only the article with results was included. Social prescribing link workers were defined as people who liaise between clients, health professionals and community organisations to connect people to community-based support, including activities and services that meet practical, social and emotional needs [13].

**Table 1:** Inclusion and exclusion criteria.

Inclusion criteria	Exclusion criteria
Primary research and clinical trial protocols	Other publication types including review articles, books, conference proceedings, editorials, and clinical guidelines
English	Non-English
Australian settings	Non-Australian setting
Social prescription referrals to non-medical services	Referral to exclusively medical services
	No link worker/connectors

### Data extraction

A data extraction tool was developed in Microsoft Word [32] based on the template by Silver and Francis [33]. Study details were systematically extracted, summarised, and displayed to facilitate comparison and synthesis [29]. Data items were: Citation, Aim, Design, Setting, Sample, Intervention, Comparator, Domains, Outcome Measures, and Findings (Table 2). Data was extracted independently by two authors (TT, CT) and inconsistencies were resolved through team discussion. For qualitative studies, an additional two authors (CA, JB) also reviewed extracted Domains and Outcome Measures to ensure accuracy of interpretation.

**Table 2:** Characteristics of included studies.

Study	Aim	Design/Setting/Participants	Social Prescribing Strategy	Intervention/Comparator	Outcome domain/s	Outcome measure/s	Findings	MMAT
**Aggar, Caruana** [46] **Social prescribing as an intervention for people with work-related injuries and psychosocial difficulties in Australia**	To describe the economic, social, health service utilisation, and quality of life outcomes of injured workers.	Mixed methods i.e., questionnaires and interviews. Sydney, Australia. *n* = 175, 18–65 years. Unable to return to work after a work-related injury acquired 6 months– 3 years prior or returned to work on reduced hours/duties.	GP identifies as experiencing psychosocial difficulties and is likely to benefit from increased social participation. Referred to care coordination service for holistic needs assessment, care planning, linkage and referral with follow-up contact.	12-week program. Follow-up post-intervention). Activities included arts and crafts, yoga and relaxation, equine therapy, and social groups. Additional referrals to financial and housing support, relationship counselling, and mental health support groups.	Global Well-Being and Needs	Camberwell Assessment of Needs Short Appraisal Schedule (CANSAS). WHO Quality of Life Brief Assessment (WHOQoL-Brief).	Social prescribing is effective in improving the overall well-being of injured workers with psychological difficulties. Benefits included increased social connectedness, confidence and ability to return to work, and reduced pain, distress, and health service needs.	4
					Social Well-Being.	The UCLA 3-item Loneliness Scale (UCLA-3). Number of people participants could count on. Satisfaction with Social Support.		
					Emotional Well-Being.	10-item Kessler Psychological Distress Scale (K10).		
					Physical Well-Being.	Pain Rating Scale (1 item, rated 0–10). EQ-5D-5L Health Thermometer (EQ5D).		
					Patient/Service User Experience	Program satisfaction ratings. Interview data.		
					Social Determinants of Health	Confidence in returning to work.		
					Health Service Utilisation	Frequency of hospitalisations and other health services. Interview data.		
					Occupational/Economic Participation.	Capacity for work. Current employment. Interview data.		
					Feasibility	Frequency of current volunteering.		
**Aggar, Thomas** [47] **Social prescribing for individuals living with mental illness in an Australian community setting: a pilot study**	Improve quality of life, and social and economic participation of people with diagnosed mental illness.	Exploratory, quantitative, longitudinal design. Sydney, Australia. *N* = 13, 18–65 years. Living in the community in the Sydney Local Health District. Diagnosed with serious mental illness likely to last 6 months or longer.	GP identifies unmet biopsychosocial needs and enrols in the program. Link workers conduct holistic needs assessment, care planning, linkage, and referral with follow-up contact.	10-week program. Follow-up six months post-baseline. All participants attend weekly arts and crafts groups (2–3 hours). Additional referrals to chronic disease management, acute care ‘hospital in the home’, financial and housing support, relationship counselling, and mentoring programs.	Global Well-Being and Needs	Global Quality of Life—WHOQoL-Brief. Camberwell Assessment of Needs Short Appraisal Schedule (CANSAS).	Significant improvement in physical and psychological QoL, health satisfaction and self-perceived health status. No significant differences in social participation self-rated loneliness, and economic participation.	4
					Social Well-Being.	UCLA 3-item Loneliness Scale.		
					Emotional Well-Being.	The Kessler Psychological Distress Scale (K10).		
					Physical Well-Being	EuroQol Health Thermometer EQ5D.		
					Economic Return	Participation in paid employment (yes/no) in the previous 2 weeks.		
**Dingle, Sharman** [48] **A controlled evaluation of social prescribing on loneliness for adults in Queensland: 8-week outcomes**	Improve loneliness and wellbeing and decrease health service usage among people experiencing loneliness or social isolation.	Non-randomised control trial. QLD, Australia *n* = 114, > = 18 years. Experiencing loneliness or social isolation based on self-report and/or identified by their health or social care workers. Frequent GP attenders (> = 12 visits per year for 2 years).	Referrals from GPs and hospitals (*n* = 20), community services (*n* = 20), and self/family referrals (*n* = 13). Link worker refers to community group activities.	8-week community group program, including art and creative activity, physical and outdoor activity, educational courses, and others). GP Treatment as usual (TAU). Post intervention follow up (8-weeks).	Social Well-Being.	8-item UCLA Loneliness Scale (ULS-8). Social anxiety: 3-item Social Phobia Inventory (mini-SPIN). Social trust: adapted version of the Cognitive Trust in Service Relationships Scale.	Improvements in loneliness, social trust, wellbeing were significantly different for social prescribing group. Psychological distress and social anxiety were not significantly different for social prescribing group but had a small to medium effect size.	4
					Emotional Well-Being.	6-item Kessler Psychological Distress Scale (K6). Warwick Edinburgh Mental Wellbeing Scale.		
					Health Service Utilisation	Frequency of hospital visits. Attendance at GPs, allied health (counsellor, psychologist, psychiatrist, social worker), and community mental health services.		
					Economic Return	Frequency of work (past month).		
					Feasibility	Percentage of participants retained at the 8-week assessment period.		
**Study Protocols**								
**Thomas, Baker** [50] **Stepped-wedge cluster randomised trial of social prescribing of forest therapy for quality of life and biopsychosocial wellbeing in community-living Australian adults with mental illness: protocol**	To improve the quality of life and biopsychosocial wellbeing of community-living adults with diagnosed severe mental illness.	Stepped-wedge cluster randomised design. Sydney-Gold Coast, Australia. *n* = 140 (planned), > = 18 years. Diagnosed with severe and persistent/complex mental illness (mood or psychotic disorder).	GP referral to care coordination service (PCCS) where link workers complete a holistic needs assessment and enrol participants. Participants also receive usual care including referral to other health and welfare services.	Initial 10-week control period. Follow-up post-intervention and 5 weeks post-intervention. 10 weekly 90-minute forest therapy sessions in groups of 6–10.	Global Well-Being and Needs	Global Quality of Life—WHOQoL-Brief.	N/A	N/A
					Social Well-Being	UCLA 3-item Loneliness Scale. Work and Social Adjustment Scale.		
					Emotional Well-Being.	Depression—Patient Health Questionnaire-9. Anxiety—Generalised Anxiety Disorder Questionnaire.		
					Physical well-being	The Health Confidence Score. Physical Health Subscale of WHO-QoL-Brief.		
					Social Determinants of Health	Work and Social Adjustment Scale.		
					Health Service Utilisation	Frequency of ambulance use, hospital visits and admissions. Nights spent in hospital. GP visits. Allied health, and community health service utilisation.		
**Jayasinghe, Holloway** [49] **An Ounce of Prevention is Worth a Pound of Cure”: Proposal for a Social Prescribing Strategy for Obesity Prevention and Improvement in Health and Well-being**	Develop sustainable community program to prevent obesity and related lifestyle diseases and enhance wellbeing among community dwelling residents of Circular Head, TAS.	Prospective multi method design i.e., questionnaires, focus groups, health system data, allied health data. Australia. Circular Head, TAS. Convenience sample.	Recruitment from community-level lifestyle screening at local events (e.g. sporting events) and workplaces, GPs and allied health, and trainee health professionals (e.g. nutrition and exercise science). Link worker assessment and referral including coproducing health goals and action plans.	3-year pilot phase. No specified follow-up timeline. Peer education, health screening, service access, and workforce connectivity. Improve food literacy, physical literacy and activity levels, mental health, community connectedness, and reduction of social isolation.	Global Well-Being and Needs	Subjective Well-Being—not specified. Quality of Life—not specified.	Circular Head residents will co-design a sustainable solution to health and wellbeing challenges and increased access to peer and other support workers.	N/A.
					Social Well-Being.	Development of Social Networks (not specified). Social return on investment (not specified).		
					Physical Well-Being.	Physiological changes (not specified). Medication usage (not specified).		
					Patient/Service User Experience	Behaviour Change—not specified.		
					Health Service Utilisation.	Frequency of Health Service Access.		
**Woolfenden** [51] **Equity Pathways in Integrated Care in Cerebral Palsy (EPIC-CP): a pilot clinical trial of social prescribing for children and young people with cerebral palsy and their parents/caregivers**	To assess the social prescribing program implementation and its health outcomes among parents of cerebral palsy patients.	Randomised control trial. NSW, ACT, Australia. *n* = 120, parents/caregiver of a child (0–18 years) with cerebral palsy, who is a patient of one of six predetermined tertiary Paediatric Rehabilitation Departments. Report at least one unmet social need from the following: Childcare or schooling; Government benefits and vouchers; Housing; Food; Bills; Transport.	Participants in the social prescribing group will receive a resource pack and be allocated a link worker.	Intervention duration unknown. Follow up at 3 months and 6 months post-randomisation. Link workers will consider family needs to refer a case-by-case intervention. Comparator unknown.	Social Well-Being	Unmet Social Needs—adapted WECARE tool.	N/A.	N/A.
					Emotional Well-Being	K-6 Distress Scale.		
					Patient/Service User Experiences.	Participants experiences—not specified. Barriers and enablers to social prescribing. PROMIS Scale. PROMIS Parent Proxy Scale PROMIS Paediatric Scale for children/young people >8 years who can self-report.		
					Feasibility	Recruitment rates. Uptake of intervention. Follow-up of participants.		
					Fidelity	Type of social prescribing activities referrals, inquires and attendance.		

Note: NSW = New South Wales, QLD = Queensland, TAS = Tasmania.

### Quality appraisal

The MMAT [30] was used to critically appraise the methodological quality across qualitative, randomized controlled, nonrandomized, quantitative descriptive, and mixed methods designs [30]. Appraisal was conducted independently by two authors (TT, CT) and a consensus approach was used to confirm the ratings according to the five core quality criteria of the MMAT [30]. Studies were scored on a scale ranging from 1 (no criteria met) to 5 (all criteria met), where higher scores indicate greater methodological rigour. The impacts of risk of publication bias (selective non-reporting) and certainty (validity and reliability) were considered and deemed as minimal. The primary aim of this integrative review was to identify and integrate the domains and measures used in studies, rather than the results. Thus, publication bias towards positive results or lack of certainty of results was not likely to significantly impact the outcomes of this integrative review.

### Synthesis of data

Studies were classified and grouped according to the type of evidence (qualitative/quantitative) followed by primary outcome. Common findings across studies were identified and colour-coded (by author TT) and re-tabulated by result. This synthesised results table was checked against full-text papers to ensure accuracy. For each study, we extracted all outcomes for which data was provided. Outcome measures were identified by name (in the case of published tools) or by focus (e.g. number of hospital visits) in the case of non-standardised outcome measures. This data was used to answer the ‘how’ of outcome measurement. To accommodate the diverse outcome terminologies used in the studies and study protocols, similar domains were grouped into categories for a more unified representation. This data was systematically coded, categorised, and summarised to identify patterns and variations across studies according to the framework and methodology for integrative reviews developed by]; these were ultimately classified into author-generated outcome domains to answer the ‘what’ of measurement focus.

## Results

**Fig 1:**
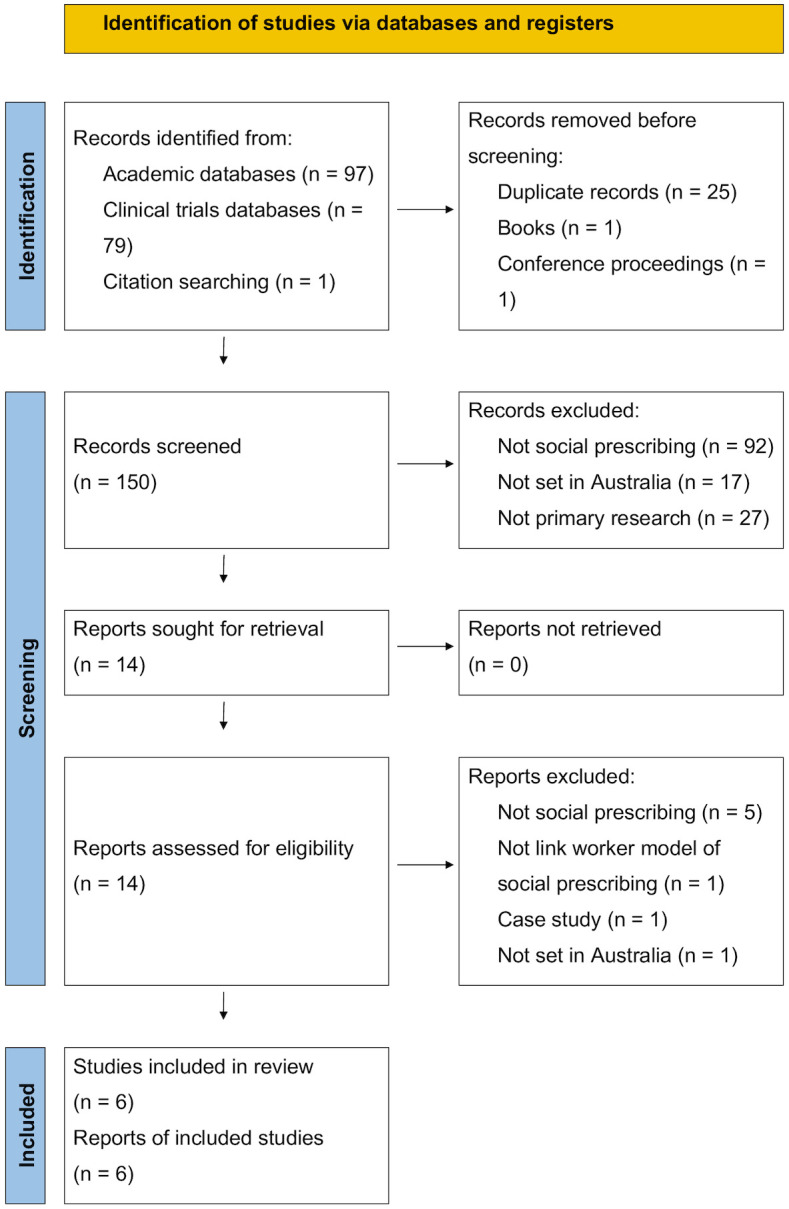
Study selection PRISMA diagram. *From: Page, McKenzie* [52].

As shown in the PRISMA diagram (Fig 1), the selection procedure initially identified 177 records from academic databases and twenty-seven records were removed, leaving 150 unique records for screening. Title and abstract screening excluded 136 records as they did not describe social prescribing interventions (*n* = 92), were not set in Australia (*n* = 17), or did not describe primary research, including reviews (*n* = 14), and other article types (*n* = 13), for example, commentaries and clinical management guidelines. Seventeen review papers’ reference lists were scanned including [1, 15, 17, 21, 22, 34–45]. Fourteen full-text reports were retrieved (all were available) and assessed for eligibility. Seven reports were excluded as they were not social prescribing (*n* = 5), did not include the link worker model of social prescribing (*n* = 1), was a case study (*n* = 1), and were not set in Australia (*n* = 1). Six studies were eligible, including three published studies [46–48], and three study protocols [49–51]. A summary of the papers comprised in this integrative review and their corresponding MMAT score are displayed in Table 2.

### Analysis of studies

The integrative review identified six eligible published studies. Three studies investigated the impact of social prescribing programs in Sydney [46, 47] or Brisbane (including regional outskirts) [48]. Three study protocols described plans for social prescribing provision in New South Wales and Queensland [50], New South Wales and the Australian Capital Territory [51], and Tasmania [49]. Participants included people with work-related injuries and psychosocial difficulties *n* = 175 [46], adults experiencing loneliness or social isolation *n* = 114 [48], and adults with mental illness *n* = 13 [47]. Proposed participants included people with obesity [49], parents/caregivers of children with Cerebral Palsy [51], and adults with mental illness [50]. Referrals to social prescribing programs were from GPs [46, 47], hospitals [48], community services [48], and self or family [48]. The study protocols proposed referrals by GPs [49, 50], allied health services [49], the community through screening at local events [49], workplaces [49], and NSW tertiary paediatric rehabilitation departments [51]. All studies were published between 2020 and 2022.

### Methodological approaches and evaluation designs

Study designs included a mixed methods design [46], an exploratory longitudinal design [47], and a non-randomised controlled design [48]. Proposed study designs included a stepped-wedge cluster randomised design [50], a prospective multi-method design [49], and a randomised controlled trial [51]. The duration between baseline and follow-up for studies was eight weeks [48], 12 weeks [46], and six months [47]. Study protocols proposed three and six-month follow-up [51], pre- and post-control, 5-weeks post-intervention, 10 weeks post-intervention follow-up [50], or did not specify measurement timelines [49].

### Outcome domains and measures

Ten outcome domains were identified among the included studies and these were categorised as ‘person-level’ (*n* = 6), ‘system-level’ (*n* = 2), and ‘program implementation’ domains (*n* = 2) (see Table 3). Two studies [46, 48] included all three domains, one study [47] and two study protocols [49, 50] included ‘person-level’ and ‘system-level’ domains, and one study protocol [51] included ‘person-level’ and ‘program implementation’ domains. The primary outcome across all studies and protocols was the person-level domain, in which global well-being, social and emotional well-being were most commonly evaluated, with the WHO Quality of Life Brief Assessment and the short form UCLA Loneliness Scale the most commonly applied standardised outcome measures. At the system level, health service utilisation was mainly evaluated (see Table 3).

**Table 3:** Summary of outcome domains and measurements.

Outcome Domains	Number of Studies	Outcome measures implemented	Outcome measure frequency (studies)
**Person-Level Domains**			
Global Well-Being and Needs	6	WHO Quality of Life Brief Assessment (WHOQOL-BREF).	3 (46, 47, 50)
*Overall quality of life and subjective well-being*, *including the perception of one’s needs being met*.			
		Quality of life—not specified.	1 (49)
		Subjective Well-Being—not specified.	1 (48, 51)
		Camberwell Assessment of Needs Short Appraisal Schedule (CANSAS).	2 (46, 47)
Social Well-Being	6	Number of people participants could count on.	1 (46)
*The quality and quantity of social support*, *social anxiety*, *trust in relationships*, *unmet social needs*, *and feelings of loneliness in individuals*.			
		Satisfaction with Social Support—not specified.	1 (46)
		3-item Social Phobia Inventory (mini-SPIN).	1 (48)
		Adapted version of the Cognitive Trust in Service Relationships Scale.	1 (48)
		Unmet Social Needs—adapted WECARE tool.	1 (51)
		UCLA Loneliness Scale (UCLA-3).	3 (46, 47, 50)
		UCLA Loneliness Scale (ULS-8).	1 (48)
		Development of Social Networks—not specified.	1 (49)
Emotional Well-Being	5	Kessler Psychological Distress Scale (K10).	2 (46, 47)
*Low levels of psychological distress*, *anxiety and depressive symptoms*. *Higher degree of positive mental health and functioning*.		Kessler Psychological Distress Scale (K6).	2 (48) (51)
		Warwick Edinburgh Mental Wellbeing Scale.	1 (48)
		Depression—Patient Health Questionnaire-9.	1 (50)
		Generalised Anxiety Disorder Questionnaire.	1 (50),
Physical Well-Being	4	EQ-5D-5L Health Thermometer (EQ5D).	2 (46, 47)
*The ability to perform daily activities with minimal pain and high confidence in managing one’s health*.			
		Pain Rating Scale (1 item, rated 0–10).	1 (46)
		Physical Health Subscale of WHOQOL-BREF.	1 (50)
		Physiological Changes—not specified.	1 (49)
		Health Self-Efficacy: The Health Confidence Score	1 (50)
Patient/Service User Experience	4	Behaviour change—not specified.	1 (49)
*Overall satisfaction and perception of healthcare services*, *including effectiveness*, *ease of access*, *impact on health*, *and support for behaviour change*.			
		Participants experiences—not specified.	1 (51)
		Program satisfaction ratings.	2 (46, 51)
		Interviews exploring the barriers and enablers to social prescribing.	1 (51)
		Patient Reported Outcomes Measurement Information System (PROMIS Scale).	1 (48, 49)
		Patient Reported Outcomes Measurement Information System (PROMIS) Parent Proxy Scale.	1 (51)
		Patient Reported Outcomes Measurement Information System (PROMIS) Paediatric Scale for children/young people >8 years who can self-report.	1 (51)
Social Determinants of Health	2	Work and Social Adjustment Scale.	1 (50)
*The conditions affecting health outcomes*, *including daily activity performance*, *work confidence*.			
		Confidence in returning to work—not specified.	1 (46)
**System-Level Domains**			
Health Service Utilisation	5	Frequency of access of other health services.	3 (46, 48, 49)
*The frequency of accessing health services*.		Frequency of hospital visits and/or hospitalisations.	3 (46, 48, 50)
		Frequency of ambulance use.	2 (46, 50)
		Frequency of nights spent in hospital.	2 (46, 50)
		Frequency of GP visits.	2 (48, 50)
		Medication Usage—not specified.	1 (49)
Economic Return	4	Capacity for work (3 categories: no, some, or full capacity).	1 (46)
*Assessment of work capacity*, *employment status*, *work frequency*, *and participation in paid employment*.			
		Current employment status (6 categories: full-time, part-time, unemployed, income support, not looking to work, worker’s compensation).	1 (46)
		Frequency of work (past month).	1 (48)
		Participation in paid employment (yes/no) in the previous 2 weeks.	1 (47)
		Social Return on Investment—not specified.	1 (49)
**Program Implementation Domains**			
Feasibility	2	Recruitment rates.	1 (51)
		Uptake of intervention.	1 (51)
		Percentage of participants retained at the 8-week assessment period.	1 (48)
Fidelity	1	Type of social prescribing activities; referrals, inquires and attendance.	1 (51)

A mixed methods study by Aggar, Caruana [46] exploring social prescribing for people with work-related injuries and psychosocial difficulties was the only study that reported on quality and interactions of the link workers. This integrative review revealed that positive relationships between participants and their link worker improved outcome measures. Woolfenden [51] is the only study that proposes to evaluate link workers through surveys and qualitative methods as part of their study design.

## Discussion

This integrative review identified only six published link worker social prescribing Australian studies, of which three were study protocols. The studies varied in their methodological approaches and study designs. Across these six papers, ten outcome domains were generated and categorised as: ‘person-level’, ‘system-level’, and ‘program implementation’ domains. Across these three outcome domains, a total of 46 outcome measures were applied. The variation in outcome domains and measures makes sense in light of the individualised approach of social prescribing but presents a challenge in terms of a core minimum dataset for the purposes of benchmarking across social prescribing programs. Overall, our findings illustrate the perceived potential for social prescribing to positively impact a range of participant groups, but there is inconsistency regarding both ‘what’ to measure and ‘how’ to measure it. This integrative review reports on the current state of evidence in Australia, with the potential to track changes and trends over time.

Despite the variation in participant groups, the primary aim in all studies was the ‘person-level’ domain, in which global well-being and social well-being were consistently evaluated. At the ‘system level’, health service utilisation was most commonly evaluated. These findings reflect international literature [44], including the recommendation for the evaluation of ‘person level’ and ‘system level’ outcome domains for social prescribing programs in the UK [41]. Future Australian studies should adopt the dual focus of ‘person level’ and ‘system level’ outcome domains to further develop the evidence base for social health provision in the context of an already stretched health system.

While global well-being and social well-being were evaluated across all studies, a variation of outcome measures were applied, the most common were the WHO Quality of Life Brief Assessment and the short form UCLA Loneliness Scale. The measurement of social well-being in this review was highly heterogeneous, a common construct and measurement challenge within the field of social connections [39]. The nine social measures identified in this integrative review provide nuanced insight into the broad range of social prescribing programs that aim to address a range of social dimensions, as one aspect of the broader social determinants of health. It is worth noting that the social determinants of health and equity are often identified as key policy drivers for funding social prescribing 36], however specific measurement of the broader social determinants of health (e.g., utilising tools like The Protocol for Responding to and Assessing Patients’ Assets, Risks, and Experiences [PRAPARE]) was under-represented in the Australian literature. While diverse outcome measures demonstrate a commitment to addressing the varied healthcare needs of different population groups and individuals, heterogeneity poses major challenges for the synthesis, comparison, and evaluation of social prescribing programs [23]. Particularly, standardised outcome measures will be required for meta-analyses, which comprehensively synthesise current evidence and are used for evidence-based decision-making in healthcare and policy [23]. Additionally, this study’s findings suggest that the current Australian evidence base is limited, which highlights the need for further research and critical assessment of the findings. Consequently, there is a need for uniformity and the use of validated measures for the core or common aims of social prescribing (e.g. quality of life) to which most programs aspire. This is essential to benchmarking and facilitating well-informed decision-making, allowing for the optimal expansion of the link worker model of social prescribing within the Australian health landscape. At the same time, there is a need for flexibility to use more specific tools that align with the more specific goals of individual programs (e.g., return to work) and inclusion of the participant’s expectation of participating in a social prescribing program.

Despite the exponential growth of research in other countries, this integrative review has identified that social prescribing in Australia is in its infancy, with only a small number of studies addressing the needs of a heterogeneous group of clients. Given the limited rollout of the link worker model of social prescribing in Australia, researchers and funders should move strategically in future application and evaluation. Recommendations for the consideration of researchers, funders and policy makers include

- A united social prescribing institute that provides opportunities for cross-disciplinary, well-resourced and consistent exploration of social prescribing, both at a policy and practice level.
- High-quality research to explore the impact and reach of social prescribing in the Australian context and encourage researchers to consider the outcome domains identified in this research to enable the amalgamation of data in future comprehensive reviews.
- Longitudinal studies, that enable exploration of outcomes and impact beyond the limit of the social prescribing program [53].
- Support from policy makers and funders to enable consistent exploration of social prescribing across the various funding models in Australia. International literature demonstrates that social prescribing has been used in hospital contexts [54, 55], community-based settings [56, 57], with children [58], people with disability [57] and older adults [38], including veterans [59, 60]. This suggests that opportunities exist in Australia across federally funded health care, state-funded healthcare, the National Disability Insurance Scheme, My Aged Care and Department of Veteran Affairs to explore social prescribing initiatives and evaluation.
- Research investigating the mechanisms of ‘how’ social prescribing works (e.g., the theoretical mechanisms of change), as well as the relational aspects of the approach which may influence how effective it is (e.g., the quality of the relationship with the link worker).
- A core set of outcome domains and measures to support benchmarking is required to be developed by Australian researchers, healthcare workers/referrers, link workers, funding bodies and consumer contribution to ensure alignment with the Australian healthcare landscape.
- The outcome measures across all studies included in this research were selected by the research team. A risk of such an approach is that outcome domains and measures may not hold meaning for participants receiving social prescribing [61]. Consequently, the addition of specific tools that align with the more specific study goals, should also clearly articulate participant consultation in determining an individual’s expectation [62].

### Strengths and limitations

This integrative review provides a comprehensive synthesis and evaluation of published research on outcome domains and measures, and the methodological approaches and evaluation designs of Australian link worker social prescribing programs. The exclusion of grey literature may be seen as a limitation. It was not in the scope of this integrative review to analyse implementation evaluation of social prescribing programs, however authors acknowledge this evidence is important for future studies to consider. As with all reviews, it is plausible that evidence may have been missed, especially in the light of the rapid expansion of this topic. Whilst this integrative literature review did not aim to target any specific cultural group, we acknowledged that the discourse around social support for Indigenous and culturally diverse communities needs to feature in social prescribing in Australia. The results are also potentially biased by types of funding supporting current link worker models of social prescribing programs, especially as many of these programs are implemented by health services without the resources to evaluate or publish. The broad range of methodologies across included studies may result in lack of rigour, inaccuracy, and bias [29]. However these potential limitations were addressed by closely following the recommended procedures for integrative reviews including duplicate independent screening and data extraction, and use of the mixed methods appraisal tool [30].

## Conclusion

In Australia, primary healthcare systems are at capacity, and social prescribing is an internationally recognised approach that has been used to address the health and social needs of individuals. The results of this research indicate that multiple opportunities exist for social prescribing across the broad range of Australian funding models and that both ‘person-level’ and ‘system-level’ domains are critical for social prescribing evaluation. The development of consumer partnerships to develop a core outcome set that can guide both national and international practice and policy is required. Lastly, in addition to the use of specific tools that align with specific study goals, there is a need for future research to clearly articulate participant consultation in determining an individual’s expectation and preferred outcomes of participating in a social prescribing program.

## Supporting information

S1 Data (XLSX)

S1 File (DOCX)

S2 File (DOCX)

S3 File (PDF)
