# Supplementary material for: Australian link worker social prescribing programs: An integrative review
Source: PLoS One. 2024 Nov 11;19(11):e0309783. doi: 10.1371/journal.pone.0309783 (PMC11554121; doi:10.1371/journal.pone.0309783)
Supplement: S1 File — (DOCX) [file pone.0309783.s002.docx]

# Search Overview

|  | **Concept** | **Search terms** | **n** |
| --- | --- | --- | --- |
| 1. | Social prescribing | "Social Prescri*"[All Fields] |  |
| 2. | Australia | "Australia"[MeSH Terms] OR "Australia*"[All Fields] |  |
| 3. | Combining searches | 1 AND 2 |  |
| 4. | Limits | English language  Peer-reviewed journal articles |  |
| - | Total (added to endnote) | Y/N |  |
| - | Full Search |  |  |

# Search 1 _ 15 July 2023

## EBSCO Psychology and Behavioral Sciences Collection

*Includes Academic Search Premier, APA PsycArticles, APA PsycINFO, CINAHL, Health Source: Nursing/Academic Edition, Humanities International Complete*

| **Step** | **Concept** | **Search terms** | **n** |
| --- | --- | --- | --- |
| 1. | Social prescribing | "Social Prescri*" | 1,014 |
| 2. | Australia | “Australia*” | 1,682,412 |
| 3. | Combining searches | 1 AND 2 | 59 |
| 4. | Limits | Peer-reviewed journal articles | 54 |
| - | Total (added to endnote) | Y | 54 |
|  | Journals | Academic Search Premier; CINAHL Plus with Full Text; Health Source: Nursing/Academic Edition; Humanities International Complete; APA PsycArticles; Psychology and Behavioral Sciences Collection; APA PsycInfo |  |
| - | Full Search | "Social Prescri*" AND "Australia*"  (limit to peer reviewed) |  |

## Cochrane Library

| **Step** | **Concept** | **Search terms** | **n** |
| --- | --- | --- | --- |
| 1. | Social prescribing | Social NEXT Prescri* | 3 |
| 2. | Australia | Australia* | 273 |
| 3. | Combining searches | 1 AND 2 | 3 |
| 4. | Limits | English language | 3 |
| - | Total (added to endnote) | Y | 3 |
| - | Full Search | (Social NEXT Prescri*) AND Australia* |  |

## Ovid (all journals)

| **Step** | **Concept** | **Search terms** | **n** |
| --- | --- | --- | --- |
| NOTE |  | *Must change resources selected to “search all Ovid journals”* |  |
| 1. | Social prescribing | "Social Prescri*".ti,ab,tx,kw.  (Title, abstract, full-text, keywords) | 654 |
| 2. | Australia | "Australia*".ti,ab,kw.  (Title, abstract, keywords) | 69,105 |
| 3. | Combining searches | 1 AND 2 | 16 |
| 4. | Limits | Limits - None | 16 |
| - | Total (added to endnote) | Y | 16 |
| - | Full Search | "Social Prescri*".ti,ab,tx,kw. and "Australia*".ti,ab,kw. |  |

## Proquest

| **Step** | **Concept** | **Search terms** | **n** |
| --- | --- | --- | --- |
|  | *Note* | *Starting with all databases selected* |  |
| 1. | Social prescribing | "Social Prescri*" | 6786 |
| 2. | Australia | Australia* | 53,127,470 |
| 3. | Combining searches | 1 AND 2 | 1155 |
| 4. | Limits | Peer reviewed  Scholarly journals  English  NOT (Literature Review AND Commentary AND Editorial AND Review AND Correspondence AND General Information AND Interview AND News)  Location: Australia  NOT (Engineering Database AND ABI/INFORM Collection AND ABI/INFORM Global AND Asian & European Business Collection AND Agriculture Science Database AND Materials Science Database AND Advanced Technologies & Aerospace Database AND Biological Science Database AND Career & Technical Education Database AND Research Library: The Arts AND Research Library: Business AND Research Library: History AND Research Library: Literature & Language AND Research Library: Science & Technology AND Career & Technical Education Database: Health & Medicine AND Middle East & Africa Database) | 21 |
| 5. |  | Show results outside my library's subscription | 23 |
| - | Total (added to endnote) | Y | 23 |
| - | Full Search |  |  |

## PubMed

| **Step** | **Concept** | **Search terms** | **n** |
| --- | --- | --- | --- |
| 1. | Social prescribing | "Social Prescri*"[All Fields] | 437 |
| 2. | Australia | "Australia"[MeSH Terms] OR "Australia*"[All Fields] | 870,165 |
| 3. | Combining searches | 1 AND 2 | 30 |
| 4. | Limits | English language  Peer-reviewed journal articles | 30 |
| - | Total (added to endnote) | Y | 30 |
| - | Full Search | "social prescri*"[All Fields] AND ("Australia"[MeSH Terms] OR "australia*"[All Fields]) |  |

## Aushealth

Search conducted as per search overview. All papers already identified in previous searches.

## SCOPUS

Search conducted as per search overview. All papers already identified in previous searches.

## Web of Science

Search conducted as per search overview. All papers already identified in previous searches.

# Updated Search 2 _ 25 October 2023

## EBSCO Psychology and Behavioral Sciences Collection

*Includes Academic Search Premier, APA PsycArticles, APA PsycINFO, CINAHL, Health Source: Nursing/Academic Edition, Humanities International Complete*

Search conducted as above. No new papers identified.

## Cochrane Library

Search conducted as above. No new papers identified.

## Ovid (all journals)

Search conducted as above. No new papers identified.

## Proquest

Search conducted as above. No new papers identified.

## PubMed

Search conducted as above. No new papers identified.

## Aushealth

Search conducted as above. No new papers identified.

## SCOPUS

Search conducted as above. No new papers identified.

## Web of Science

Search conducted as above. No new papers identified.
