# Supplementary material for: Australian link worker social prescribing programs: An integrative review
Source: PLoS One. 2024 Nov 11;19(11):e0309783. doi: 10.1371/journal.pone.0309783 (PMC11554121; doi:10.1371/journal.pone.0309783)
Supplement: S2 File — (DOCX) [file pone.0309783.s003.docx]

| **Study** | **Aim** | **Design/Setting/Participants** | | **Social Prescribing Strategy** | **Intervention/Comparator** | **Outcome domain/s** | **Outcome measure/s** | **Findings** | **MMAT** |
| --- | --- | --- | --- | --- | --- | --- | --- | --- | --- |
| **Aggar, Caruana (46)**  **Social prescribing as an intervention for people with work-related injuries and psychosocial difficulties in Australia** | To describe the economic, social, health service utilisation, and quality of life outcomes of injured workers. | Mixed methods i.e., questionnaires and interviews.  Sydney, Australia.  *n* = 175, 18–65 years.  Unable to return to work after a work-related injury acquired 6 months – 3 years prior or returned to work on reduced hours/duties. | | GP identifies as experiencing psychosocial difficulties and is likely to benefit from increased social participation.  Referred to care coordination service for holistic needs assessment, care planning, linkage and referral with follow-up contact. | 12-week program.  Follow-up post-intervention).  Activities included arts and crafts, yoga and relaxation, equine therapy, and social groups.  Additional referrals to financial and housing support, relationship counselling, and mental health support groups. | Global Well-Being and Needs | Camberwell Assessment of Needs Short Appraisal Schedule (CANSAS).  WHO Quality of Life Brief Assessment (WHOQoL-Brief). | Social prescribing is effective in improving the overall well-being of injured workers with psychological difficulties.  Benefits  included increased social connectedness, confidence and  ability to return to work, and reduced pain, distress, and  health service needs. | 4 |
|  |  |  |  |  |  | Social Well-Being. | The UCLA 3-item Loneliness Scale (UCLA-3).  Number of people participants could count on.  Satisfaction with Social Support. |  |  |
|  |  |  |  |  |  | Emotional Well-Being. | 10-item Kessler Psychological Distress Scale (K10). |  |  |
|  |  |  |  |  |  | Physical Well-Being. | Pain Rating Scale (1 item, rated 0 – 10).  EQ-5D-5L Health Thermometer (EQ5D). |  |  |
|  |  |  |  |  |  | Patient/Service User Experience | Program satisfaction ratings.  Interview data. |  |  |
|  |  |  |  |  |  | Social Determinants of Health | Confidence in returning to work. |  |  |
|  |  |  |  |  |  | Health Service Utilisation | Frequency of hospitalisations and other health services.  Interview data. |  |  |
|  |  |  |  |  |  | Occupational/Economic Participation. | Capacity for work.  Current employment.  Interview data. |  |  |
|  |  |  |  |  |  | Feasibility | Frequency of current volunteering. |  |  |
| **Aggar, Thomas (47)**  **Social prescribing for individuals living with mental illness in an Australian community setting: a pilot study** | Improve quality of life, and social and economic participation of people with diagnosed mental illness. | Exploratory, quantitative, longitudinal design.  Sydney, Australia.  *N* = 13, 18–65 years.  Living in the community in the Sydney Local Health District.  Diagnosed with serious mental illness likely to last 6 months or longer. | | GP identifies unmet biopsychosocial needs and enrols in the program.  Link workers conduct holistic needs assessment, care planning, linkage, and referral with follow-up contact. | 10-week program.  Follow-up six months post-baseline.  All participants attend weekly arts and crafts groups (2–3 hours).  Additional referrals to chronic disease management, acute care ‘hospital in the home’, financial and housing support, relationship counselling, and mentoring programs. | Global Well-Being and Needs | Global Quality of Life—WHOQoL-Brief.  Camberwell Assessment of Needs Short Appraisal Schedule (CANSAS). | Significant improvement in physical and  psychological QoL, health satisfaction and self-perceived  health status.  No significant differences in social  participation self-rated loneliness, and economic participation. | 4 |
|  |  |  |  |  |  | Social Well-Being. | UCLA 3-item Loneliness Scale. |  |  |
|  |  |  |  |  |  | Emotional Well-Being. | The Kessler Psychological Distress Scale (K10). |  |  |
|  |  |  |  |  |  | Physical Well-Being | EuroQol Health Thermometer EQ5D. |  |  |
|  |  |  |  |  |  | Economic Return | Participation in paid employment (yes/no) in the previous 2 weeks. |  |  |
| **Dingle, Sharman (48)**  **A controlled evaluation of social prescribing on loneliness for adults in Queensland: 8-week outcomes** | Improve loneliness and wellbeing and decrease health service usage among people experiencing loneliness or social isolation. | Non-randomised control trial.  QLD, Australia  *n* = 114, >=18 years.  Experiencing loneliness or social isolation based on self-report and/or identified by their health or social care workers.  Frequent GP attenders (>= 12 visits per year for 2 years). | | Referrals from GPs and hospitals (*n* = 20), community services (*n* = 20), and self/family referrals (*n* = 13).  Link worker refers to community group activities. | 8-week community group program, including art and creative activity, physical and outdoor activity, educational courses, and others).  GP Treatment as usual (TAU).  Post intervention follow up (8-weeks). | Social Well-Being. | 8-item UCLA Loneliness Scale (ULS-8).  Social anxiety: 3-item Social Phobia Inventory (mini-SPIN).  Social trust: adapted version of the Cognitive Trust in Service Relationships Scale. | Improvements in loneliness, social trust, wellbeing were significantly different for social prescribing group.  Psychological distress and social anxiety were not significantly different for social prescribing group but had a small to medium effect size. | 4 |
|  |  |  |  |  |  | Emotional Well-Being. | 6-item Kessler Psychological Distress Scale (K6).  Warwick Edinburgh Mental Wellbeing Scale. |  |  |
|  |  |  |  |  |  | Health Service Utilisation | Frequency of hospital visits.  Attendance at GPs, allied health (counsellor, psychologist, psychiatrist, social worker), and community mental health services. |  |  |
|  |  |  |  |  |  | Economic Return | Frequency of work (past month). |  |  |
|  |  |  |  |  |  | Feasibility | Percentage of participants retained at the 8-week assessment period. |  |  |
| **Study Protocols** | | | | | | | | | |
| **Thomas, Baker (50)**  **Stepped-wedge cluster randomised trial of social prescribing of forest therapy for quality of life and biopsychosocial wellbeing in community-living Australian adults with mental illness: protocol** | To improve the quality of life and biopsychosocial  wellbeing of community-living adults with diagnosed severe mental illness. | Stepped-wedge  cluster randomised design.  Sydney-Gold Coast, Australia.  *n* = 140 (planned),  >=18 years.  Diagnosed with severe and persistent/complex mental illness (mood or psychotic disorder). | GP referral to care coordination service (PCCS) where link workers complete a holistic needs assessment and enrol participants.  Participants also receive usual care including referral to other health and welfare services. | | Initial 10-week control period.  Follow-up post-intervention and 5 weeks post-intervention.  10 weekly 90-minute forest therapy sessions in groups of 6–10. | Global Well-Being and Needs | Global Quality of Life—WHOQoL-Brief. | N/A | N/A |
|  |  |  |  |  |  | Social Well-Being | UCLA 3-item Loneliness Scale.  Work and Social Adjustment Scale. |  |  |
|  |  |  |  |  |  | Emotional Well-Being. | Depression—Patient Health Questionnaire-9.  Anxiety—Generalised Anxiety Disorder Questionnaire. |  |  |
|  |  |  |  |  |  | Physical well-being | The Health Confidence Score.  Physical Health Subscale of WHO-QoL-Brief. |  |  |
|  |  |  |  |  |  | Social Determinants of Health | Work and Social Adjustment Scale. |  |  |
|  |  |  |  |  |  | Health Service Utilisation | Frequency of ambulance use, hospital visits and admissions. Nights spent in hospital.  GP visits.  Allied health, and community health service utilisation. |  |  |
| **Jayasinghe, Holloway (49)**  **An Ounce of Prevention is Worth a Pound of Cure”: Proposal for a Social Prescribing Strategy for Obesity Prevention and Improvement in Health and Well-being** | Develop sustainable community program to prevent obesity and related lifestyle diseases and enhance wellbeing among community dwelling residents of Circular Head, TAS. | Prospective multi method design i.e., questionnaires, focus groups, health system data, allied health data.  Australia.  Circular Head,  TAS.  Convenience sample. | Recruitment from community-level lifestyle screening at local events (e.g. sporting events) and workplaces, GPs and allied health, and trainee health professionals (e.g. nutrition and exercise science).  Link worker assessment and referral including coproducing health goals and action plans. | | 3-year pilot phase.  No specified follow-up timeline.  Peer education, health screening, service access, and workforce connectivity.    Improve food literacy, physical literacy and activity levels, mental health, community connectedness, and reduction of social isolation. | Global Well-Being and Needs | Subjective Well-Being - not specified.  Quality of Life - not specified. | Circular Head residents will co-design a sustainable solution to health and wellbeing challenges and increased access to peer and other support workers. | N/A. |
|  |  |  |  |  |  | Social Well-Being. | Development of Social Networks (not specified).  Social return on investment (not specified). |  |  |
|  |  |  |  |  |  | Physical Well-Being. | Physiological changes (not specified).  Medication usage (not specified). |  |  |
|  |  |  |  |  |  | Patient/Service User Experience | Behaviour Change - not specified. |  |  |
|  |  |  |  |  |  | Health Service Utilisation. | Frequency of Health Service Access. |  |  |
| **Woolfenden (51)**  **Equity Pathways in Integrated Care in Cerebral Palsy (EPIC-CP): a pilot clinical trial of social prescribing for children and young people with cerebral palsy and their parents/caregivers** | To assess the social prescribing program implementation and its health outcomes among parents of cerebral palsy patients. | Randomised control trial.  NSW, ACT, Australia.  *n* = 120, parents/caregiver of a child (0-18 years) with cerebral palsy, who is a patient of one of six predetermined tertiary Paediatric Rehabilitation Departments.  Report at least one unmet social need from the following: Childcare or schooling; Government benefits and vouchers; Housing; Food; Bills; Transport. | Participants in the social prescribing group will receive a resource pack and be allocated a link worker. | | Intervention duration unknown.  Follow up at 3 months and 6 months post-randomisation.  Link workers will consider family needs to refer a case-by-case intervention.  Comparator unknown. | Social Well-Being | Unmet Social Needs - adapted WECARE tool. | N/A. | N/A. |
|  |  |  |  |  |  | Emotional Well-Being | K-6 Distress Scale. |  |  |
|  |  |  |  |  |  | Patient/Service User Experiences. | Participants experiences – not specified.  Barriers and enablers to social prescribing.  PROMIS Scale.  PROMIS Parent Proxy Scale  PROMIS Paediatric Scale for children/young people >8 years who can self-report. |  |  |
|  |  |  |  |  |  | Feasibility | Recruitment rates.  Uptake of intervention.  Follow-up of participants. |  |  |
|  |  |  |  |  |  | Fidelity | Type of social prescribing activities referrals, inquires and attendance. |  |  |
