# Supplementary material for: Australian link worker social prescribing programs: An integrative review
Source: PLoS One. 2024 Nov 11;19(11):e0309783. doi: 10.1371/journal.pone.0309783 (PMC11554121; doi:10.1371/journal.pone.0309783)
Supplement: S3 File — (PDF) [file pone.0309783.s004.pdf]

Australian link worker social prescribing programs: an integrative review

Please note, there was no missing data or statistical analysis

Excluded papers: full-text review and reasons for exclusion

	Paper	Reason for exclusion
1	Barnett, A., Savic, M., Forbes, D., Best, D., Sandral, E., Bathish, R., ... & Lubman, D. I. (2022). Transitioning to civilian life: The importance of social group engagement and identity among Australian Defence Force veterans. *Australian & New Zealand Journal of Psychiatry*, *56*(8), 1025-1033.	Not a Social Prescribing intervention
2	Dias, R., Parker, J., Powell, L., Teige, C., Garside, M., & Wright, B. (2024). 'Safety Nets': a community based social prescribing intervention involving combined physical activity and psychoeducation for young people on mental health service waiting lists: a pilot service evaluation. *Advances in Mental Health*, *22*(1), 104-117.	Not set in Australia
3	Foley, H., Leach, M., Feng, X., Astell-Burt, T., & Brymer, E. (2023). Towards key principles for the design and implementation of nature prescription programs. *Sustainability*, *15*(12), 9530.	Not a Social Prescribing intervention
4	Huggins, L., Davis, M. C., Rooney, R., & Kane, R. (2008). Socially prescribed and self-oriented perfectionism as predictors of depressive diagnosis in preadolescents. *Journal of Psychologists and Counsellors in Schools*, *18*(2), 182-194.	Not a Social Prescribing intervention
5	Lawn, S., Huang, N., Zabeen, S., Smith, D., Battersby, M., Redpath, P., ... & Fairweather-Schmidt, K. (2019). Outcomes of telephone-delivered low-intensity cognitive behaviour therapy (LiCBT) to community dwelling Australians with a recent hospital admission due to depression or anxiety: MindStep™. *BMC psychiatry*, *19*, 1-16.	Not a Social Prescribing intervention
6	Ostojic, K., Paget, S., Martin, T., Dee-Price, B. J., McIntyre, S., Sheedy, H. S., ... & Woolfenden, S. (2023). Codesigning a social prescribing pathway to address the social determinant of health concerns of children with cerebral palsy and their families in Australia: a protocol for a mixed-methods formative research study. *BMJ open*, *13*(4), e066346.	Not a Social Prescribing intervention

Title/abstract review and reasons for exclusion

	Paper	Reason for exclusion
1	Actrn. (2022). Equity Pathways in Integrated Care in Cerebral Palsy (EPIC-CP): a pilot clinical trial of social prescribing for children and young people with cerebral palsy and their parents/caregivers [Trial registry record]. https://trialsearch.who.int/Trial2.aspx?TrialID=ACTRN12622001459718. https://www.cochranelibrary.com/central/doi/10.1002/central/CN-02498089/full	Inpatient
2	Al-Khudairy, L., Ayorinde, A., Ghosh, I., Grove, A., Harlock, J., Meehan, E., Briggs, A., Court, R., & Clarke, A. (2022). Health and Social Care Delivery Research. In Evidence and methods required to evaluate the impact for patients who use social prescribing: a rapid systematic review and qualitative interviews. National Institute for Health and Care Research	Review
3	Anderson, R. C. (2018). Mate copying and the effects of sexual history on romantic desirability. Evolutionary Psychological Science, 4(3), 322-330. https://doi.org/10.1007/s40806-018-0143-y	Not a Social Prescribing intervention

4	Anderst, A., Hunter, K., Andersen, M., Walker, N., Coombes, J., Raman, S., Moore, M., Ryan, L., Jersky, M., Mackenzie, A., Stephensen, J., Williams, C., Timbery, L., Doyle, K., Lingam, R., Zwi, K., Sheppard-Law, S., Erskine, C., Clapham, K., & Woolfenden, S. (2022). Screening and social prescribing in healthcare and social services to address housing issues among children and families: a systematic review. BMJ Open, 12(4), e054338. https://doi.org/10.1136/bmjopen-2021-054338	Review
5	10th International Conference on Herbal Medicine. Australian Journal of Herbal Medicine, 29(1), 2-6,8-13,15-38. https://ezproxy.scu.edu.au/login?url=https://www.proquest.com/scholarly-journals/10th-international-conference-on-herbal-medicine/docview/2038582339/se-2?accountid=16926	Conference
6	Antecol, H., & Cobb-Clark, D. A. (2008). Identity and racial harassment [Article]. Journal of Economic Behavior & Organization, 66(3/4), 529-557. https://doi.org/10.1016/j.jebo.2006.04.008	Not a Social Prescribing intervention
7	Askew, D. A., Lyall, V. J., Ewen, S. C., Paul, D., & Wheeler, M. (2017). Understanding practitioner professionalism in Aboriginal and Torres Strait Islander health: lessons from student and registrar placements at an urban Aboriginal and Torres Strait Islander primary healthcare service. Australian Journal of Primary Health, 23(5), 446-450. https://doi.org/https://doi.org/10.1071/PY16145	Not a Social Prescribing intervention
8	Bastiampillai, T., Jones, G. M., Furber, G., Moreau, M., Healey, D., Watson, J., & Battersby, M. (2014). The IAPT@Flinders Service: adapting the Improving Access to Psychological Therapies model to the emergency department setting in Australia. Australas Psychiatry, 22(3), 277-280. https://doi.org/10.1177/1039856214530016	Outpatient
9	Bild, E., & Pachana, N. A. (2022). Social prescribing: A narrative review of how community engagement can improve wellbeing in later life [Article]. Journal of Community & Applied Social Psychology, 32(6), 1148-1215. https://doi.org/10.1002/casp.2631	Review
10	Bond, C., Foley, W., & Askew, D. (2016). "It puts a human face on the researched" - A qualitative evaluation of an Indigenous health research governance model. Australian & New Zealand Journal of Public Health, 40 Supplement(1), S89-S95.	Not a Social Prescribing intervention
11	Buechner, H., Toparlak, S. M., Ostinelli, E. G., Shokraneh, F., Nicholls-Mindlin, J., Cipriani, A., Geddes, J. R., & Syed Sheriff, R. (2023). Community interventions for anxiety and depression in adults and young people: A systematic review [Article]. Australian & New Zealand Journal of Psychiatry, 1. https://doi.org/10.1177/00048674221150362	Review
12	Burns, P., & Van Der Meer, R. (2021,). Happy Hookers: findings from an international study exploring the effects of crochet on wellbeing. Perspect Public Health, 141(3), 149-157. https://doi.org/10.1177/1757913920911961	International
13	Costello, L. a., McDermott, M.-L. b., Patel, P. a., & Dare, J. a. (2019). 'A lot better than medicine' - Self-organised ocean swimming groups as facilitators for healthy ageing. Health & Place November, 60, 102212. https://ovidsp.ovid.com/ovidweb.cgi?T=JS&CSC=Y&NEWS=N&PAGE=fulltext&D=ovftu&AN=00126351-201911000-00010	Not a Social Prescribing intervention
14	Craig, H., Gasevic, D., Ryan, J., Owen, A., McNeil, J., Woods, R., Britt, C., Ward, S., & Freak-Poli, R. (2023). Socioeconomic, Behavioural, and Social Health Correlates of Optimism and Pessimism in Older Men and Women: A	Not a Social Prescribing intervention

	Cross-Sectional Study. Int J Environ Res Public Health, 20(4). https://doi.org/10.3390/ijerph20043259	
15	Dammery, G. B., Research, Administration, O., Vitangcol, K. M. P. H. M., Research, A., Ansell, J. B., Ellis, L. A. P., Research, F., Smith, C. L. P., Research, O., Carrigan, A. P., Research, F., Braithwaite, J. P., Professor, Zurynski, Y. P., & Professor. (2023). The Patient Activation Measure (PAM) and the pandemic: Predictors of patient activation among Australian health consumers during the COVID-19 pandemic. Health Expectations, 26(3), 1107-1117. https://ovidsp.ovid.com/ovidweb.cgi?T=JS&CSC=Y&NEWS=N&PAGE=fulltext&D=ovftz&AN=00126350-202305000-00012	Not a Social Prescribing intervention
16	Davies, C. R., Knuiman, M., Wright, P., & Rosenberg, M. (2014). The art of being healthy: a qualitative study to develop a thematic framework for understanding the relationship between health and the arts. BMJ Open, 4(4), e004790. https://doi.org/10.1136/bmjopen-2014-004790	Not a Social Prescribing intervention
17	Dingle, G. A., Sharman, L. S., Haslam, C., Donald, M., Turner, C., Partanen, R., Lynch, J., Draper, G., & van Driel, M. L. (2021). The effects of social group interventions for depression: Systematic review [journal article]. Journal of Affective Disorders, 281, 67-81. https://doi.org/10.1016/j.jad.2020.11.125	Review
18	Du, W., Wang, J., & Zhou, Q. (2021). Urgent Need of Integrated Health and Social Care to Alleviate High Psychological Distress in People with Disabilities: A Cross-Sectional National Representative Survey in Australia. Risk Management and Healthcare Policy, 14, 1541-1550. https://doi.org/https://doi.org/10.2147/RMHP.S291004	Inpatient
19	Eaton, G., Wong, G., Tierney, S., Roberts, N., Williams, V., & Mahtani, K. R. (2021). Understanding the role of the paramedic in primary care: a realist review. BMC Medicine, 19, 1-14. https://doi.org/https://doi.org/10.1186/s12916-021-02019-z	Review
20	Fitzmaurice, C. M. P. H., Policy, & Data Analytics, O. (2022). Social prescribing: A new paradigm with additional benefits in rural Australia. Australian Journal of Rural Health, 30(2), 298-299. https://ovidsp.ovid.com/ovidweb.cgi?T=JS&CSC=Y&NEWS=N&PAGE=fulltext&D=ovfty&AN=00075406-202204000-00018	Review
21	Foley, S. (2018). "My God! Why Was I Born?" [Article]. Journal of Family History, 43(4), 357-373. https://doi.org/10.1177/0363199018781207	Not a Social Prescribing intervention
22	Foster, D. (2018). Social prescribing and the Lindsay Leg Club model of care [Article]. British Journal of Community Nursing, 23, S42-S42. https://doi.org/10.12968/bjcn.2018.23.Sup12.S42	International
23	Gilligan, I. (2020). Identity, kinship, and the evolution of cooperation: Comment. Current Anthropology, 61(2), 206-207.	Not a Social Prescribing intervention
24	Hart, N. H., Smith, A. B., Hobbs, K., Thamm, C., Gordon, L. G., Crichton, M., & Chan, R. J. (2021). Juggling cancer and life in survivorship. Australian Journal of General Practice, 50(8), 520-525. https://ezproxy.scu.edu.au/login?url=https://www.proquest.com/scholarly-journals/juggling-cancer-life-survivorship/docview/2561528767/se-2?accountid=16926	Not a Social Prescribing intervention
25	Herrmann, W., Napierala, H., Kanehl, D., Krüger, K., & Holzinger, F. (2021). The evidence of social prescribing – Challenges found in conducting a	Review

	systematic review [Article]. European Journal of Public Health, 31, iii167-iii167. https://ezproxy.scu.edu.au/login?url=https://search.ebscohost.com/login.aspx?direct=true&db=aph&AN=153588784&site=ehost-live	
26	Hicks, J. S., Burgman, M. A., Marewski, J. N., Fidler, F., & Gigerenzer, G. (2012). Decision Making in a Human Population Living Sustainably. Conservation Biology, 26(5), 760-768. https://ovidsp.ovid.com/ovidweb.cgi?T=JS&CSC=Y&NEWS=N&PAGE=fulltext&D=ovftn&AN=00009102-201210000-00002	Not a Social Prescribing intervention
27	Htun, H. L., Teshale, A. B., Cumpston, M. S., Demos, L., Ryan, J., Owen, A., & Freak-Poli, R. (2023). Effectiveness of social prescribing for chronic disease prevention in adults: a systematic review and meta-analysis of randomised controlled trials. J Epidemiol Community Health, 77(4), 265-276. https://doi.org/10.1136/jech-2022-220247	Review
28	Hughes, J. (2011). ARE OLDER MEN TAKING SEXUALITY AS PRESCRIBED? [Article]. Australian Feminist Studies, 26(67), 89-102. https://doi.org/10.1080/08164649.2010.546330	Not a Social Prescribing intervention
29	Irwin, L., Rhodes, P., & Boydell, K. (2022, 06//). Evaluation of a gallery-based Arts Engagement program for depression [Article]. Australian Psychologist, 57(3), 186-196. https://doi.org/10.1080/00050067.2022.2061329	Not a Social Prescribing intervention
30	Islam, M. M. (2020). Social Prescribing-An Effort to Apply a Common Knowledge: Impelling Forces and Challenges. Front Public Health, 8, 515469. https://doi.org/10.3389/fpubh.2020.515469	Review
31	Ivers, R., & Astell-Burt, T. (2023). Nature Rx: Nature prescribing in general practice. Australian Journal of General Practice, 52(4), 183-187. https://ezproxy.scu.edu.au/login?url=https://www.proquest.com/scholarly-journals/nature-rx-prescribing-general-practice/docview/2797704119/se-2?accountid=16926	Review
32	Jha, M., Barrett, B., Brewin, C., Bowker, G., Harwood, N., Jalil, I., Crawford, M., Phull, J., Allen, K., Duggan, C., Yang, M., & Tyrer, P. (2022). Matching ICD-11 personality status to clinical management in a community team-The Boston (UK) Personality Project: Study protocol. Personal Ment Health, 16(2), 130-137. https://doi.org/10.1002/pmh.1544	Not a Social Prescribing intervention
33	Jones, B., Woolfenden, S., Pengilly, S., Breen, C., Cohn, R., Biviano, L., Johns, A., Worth, A., Lamb, R., Lingam, R., Silove, N., Marks, S., Tzioumi, D., & Zwi, K. (2020). COVID-19 pandemic: The impact on vulnerable children and young people in Australia. Journal of Paediatrics & Child Health, 56(12), 1851-1855. https://ovidsp.ovid.com/ovidweb.cgi?T=JS&CSC=Y&NEWS=N&PAGE=fulltext&D=ovftw&AN=00002208-202012000-00006	Not a Social Prescribing intervention
34	Kane, R., Lea, T., Murphy, D., & Pienaar, K. (2017). The future of drugs: recreational drug use and sexual health among gay and other men who have sex with men. Sexual Health (Online), 14(1), 42-50. https://doi.org/https://doi.org/10.1071/SH16080	Not a Social Prescribing intervention
35	Krause, A. E., & Davidson, J. W. (2022). An exploratory study of historical representations of love in an art gallery exhibition. Psychology of Aesthetics, Creativity, and the Arts, 16(3), 455-467. https://doi.org/https://doi.org/10.1037/aca0000391	Not a Social Prescribing intervention
36	Lawson, J. T., Wissing, R., Henderson-Wilson, C., Snell, T., Chambers, T. P., McNeil, D. G., & Nuttman, S. (2022). Health empowerment scripts:	Commentary

	Simplifying social/green prescriptions. Front Psychol, 13, 889250. https://doi.org/10.3389/fpsyg.2022.889250	
37	Leigh, J., Borwell, J., Garrow, A., Kenny, A., Knight, K. H., Monks, R., Roberts, D., Whaley, V., & Wright, K. (2022). Has the pandemic response entrenched a pathogenic emphasis in education? [Article]. British Journal of Nursing, 31(20), 1066-1067. https://doi.org/10.12968/bjon.2022.31.20.1066	Not a Social Prescribing intervention
38	Lunt, N., Bainbridge, L., & Rippon, S. (2021). Strengths, assets and place - The emergence of Local Area Coordination initiatives in England and Wales. Journal of Social Work, 21(5), 1041-1064. https://ovidsp.ovid.com/ovidweb.cgi?T=JS&CSC=Y&NEWS=N&PAGE=fulltext&D=ovftx&AN=00120259-202121050-00003	International
39	Macintyre, P. E., Roberts, L. J., & Huxtable, C. A. (2020). Management of Opioid-Tolerant Patients with Acute Pain: Approaching the Challenges. Drugs, 80(1), 9-21. https://doi.org/https://doi.org/10.1007/s40265-019-01236-4	Review
40	Mann, J., Devine, S., & Strivens, E. (2022). Primary care and the older person with complex needs: reflections on the implementation of a primary–secondary model of integrated care. Australian Journal of Primary Health, 28(6), 469-473. https://doi.org/https://doi.org/10.1071/PY21236	Review
41	McHale, S., Pearsons, A., Neubeck, L., & Hanson, C. L. (2020). Green Health Partnerships in Scotland; Pathways for Social Prescribing and Physical Activity Referral. Int J Environ Res Public Health, 17(18). https://doi.org/10.3390/ijerph17186832	International
42	McIntyre, J. C., Elahi, A., Barlow, F. K., White, R. G., & Bentall, R. P. (2021). The relationship between ingroup identity and Paranoid ideation among people from African and African Caribbean backgrounds [Article]. Psychology & Psychotherapy: Theory, Research & Practice, 94(1), 16-32. https://doi.org/10.1111/papt.12261	Not a Social Prescribing intervention
43	Morse, D. F., Sandhu, S., Mulligan, K., Tierney, S., Polley, M., Chiva Giurca, B., Slade, S., Dias, S., Mahtani, K. R., Wells, L., Wang, H., Zhao, B., De Figueiredo, C. E. M., Meijs, J. J., Nam, H. K., Lee, K. H., Wallace, C., Elliott, M., Mendive, J. M., Robinson, D., Palo, M., Herrmann, W., Østergaard Nielsen, R., & Husk, K. (2022). Global developments in social prescribing. BMJ Glob Health, 7(5). https://doi.org/10.1136/bmjgh-2022-008524	Review
44	Muhl, C., Mulligan, K., Bayoumi, I., Ashcroft, R., & Godfrey, C. (2023). Establishing internationally accepted conceptual and operational definitions of social prescribing through expert consensus: a Delphi study. BMJ Open, 13(7). https://doi.org/https://doi.org/10.1136/bmjopen-2022-070184	Not a Social Prescribing intervention
45	Munindradasa, A., Blashki, G., Dykgraaf, S. H., Desborough, J., & Kidd, M. (2021). General practitioner management of mental health during the COVID-19 pandemic. Australian Journal of General Practice, 50(7), 472-477. https://ezproxy.scu.edu.au/login?url=https://www.proquest.com/scholarly-journals/general-practitioner-management-mental-health/docview/2549299069/se-2?accountid=16926	Not a Social Prescribing intervention
46	Nguyen, P. Y., Astell-Burt, T., Rahimi-Ardabili, H., & Feng, X. (2023). Effect of nature prescriptions on cardiometabolic and mental health, and physical activity: a systematic review. Lancet Planet Health, 7(4), e313-e328. https://doi.org/10.1016/s2542-5196(23)00025-6	Review

47	Ogrin, R., Cyarto, E. V., Harrington, K. D., Haslam, C., Lim, M. H., Golenko, X., Bush, M., Vadasz, D., Johnstone, G., & Lowthian, J. A. (2021). Loneliness in older age: What is it, why is it happening and what should we do about it in Australia? Australasian Journal on Ageing, 40(2), 202-207. https://ovidsp.ovid.com/ovidweb.cgi?T=JS&CSC=Y&NEWS=N&PAGE=fulltext&D=ovftw&AN=00137609-202106000-00014	Review
48	Oster, C., Gransbury, B., Anderson, D., Martin, V., Skuza, P., & Leibbrandt, R. (2023). Development and validation of a self-report social determinants of health questionnaire in Australia. Health Promotion International June, 38, 3. https://ovidsp.ovid.com/ovidweb.cgi?T=JS&CSC=Y&NEWS=N&PAGE=fulltext&D=ovftz&AN=00013240-202306000-00068	Not a Social Prescribing intervention
49	Oster, C., Skelton, C., Leibbrandt, R., Hines, S., & Bonevski, B. (2023). Models of social prescribing to address non-medical needs in adults: a scoping review [Article]. BMC Health Services Research, 23(1), 1-20. https://doi.org/10.1186/s12913-023-09650-x	Review
50	Pavli, A., Loblay, V., Rychetnik, L., & Usherwood, T. (2023). What can we learn from Australian general practices taking steps to be more environmentally sustainable? A qualitative study. Family Practice, 40(3), 465-472. https://ovidsp.ovid.com/ovidweb.cgi?T=JS&CSC=Y&NEWS=N&PAGE=fulltext&D=ovftz&AN=00003856-202305000-00006	Not a Social Prescribing intervention
51	Redmond, J. D., Pedersen, A., & Paradies, Y. (2014). Psychosocial Predictors of Antiracist Bystander Action Toward Indigenous Australians. Peace & Conflict: Journal of Peace Psychology, 20(4), 474-490. https://ovidsp.ovid.com/ovidweb.cgi?T=JS&CSC=Y&NEWS=N&PAGE=fulltext&D=ovftp&AN=00062930-201411000-00011	Not a Social Prescribing intervention
52	Reece, L. J., Quirk, H., Wellington, C., Haake, S. J., & Wilson, F. (2019, Mar 2019). Bright Spots, physical activity investments that work: Parkrun; a global initiative striving for healthier and happier communities. British Journal of Sports Medicine, 53(6), 326. https://doi.org/https://doi.org/10.1136/bjsports-2018-100041	International
53	Sather, M. (2015). Stories Matter: A Narrative Practice Approach to Bereavement Through Suicide [Article]. Grief Matters: The Australian Journal of Grief & Bereavement, 18(3), 68-74. https://ezproxy.scu.edu.au/login?url=https://search.ebscohost.com/login.aspx?direct=true&db=aph&AN=114526621&site=ehost-live	Not a Social Prescribing intervention
54	Sharman, L. S., McNamara, N., Hayes, S., & Dingle, G. A. (2022). Social prescribing link workers—A qualitative Australian perspective. Health & Social Care in the Community, 30(6), e6376-e6385. https://doi.org/https://doi.org/10.1111/hsc.14079	Not a Social Prescribing intervention
55	Shih, P., Hallam, L., Robyn, C. W., Carter, S. M., & Brown, A. (2022). Reimagining consumer involvement: Resilient system indicators in the COVID-19 pandemic response in New South Wales, Australia. Health Expectations, 25(4), 1988-2001. https://doi.org/https://doi.org/10.1111/hex.13556	Not a Social Prescribing intervention
56	Siette, J., Taylor, N., Deckers, K., Kohler, S., Braithwaite, J., Valenzuela, M., & Armitage, C. J. (2022). Advancing Australian public health initiatives targeting dementia risk reduction. Australasian Journal on Ageing, 41(2), e190-e195.	Not a Social Prescribing intervention

	https://ovidsp.ovid.com/ovidweb.cgi?T=JS&CSC=Y&NEWS=N&PAGE=fulltext&D=ovfty&AN=00137609-202206000-00036	
57	Smith, B. J., & Lim, M. H. (2020, Jun 30). How the COVID-19 pandemic is focusing attention on loneliness and social isolation. Public Health Res Pract, 30(2). https://doi.org/10.17061/phrp3022008	Not a Social Prescribing intervention
58	Taylor, A. K., Palmer, V. J., Davidson, S., Fletcher, S., & Gunn, J. (2022). Patient reported self-help strategies and the perceived benefits for managing sub-threshold depressive symptoms: A nested qualitative study of Australian primary care attendees [Article]. Health & Social Care in the Community, 30(5), e2097-e2108. https://doi.org/10.1111/hsc.13646	Not a Social Prescribing intervention
59	Thomas, T., Aggar, C., Baker, J., Massey, D., Thomas, M., D'Appio, D., & Brymer, E. (2022). Social prescribing of nature therapy for adults with mental illness living in the community: A scoping review of peer-reviewed international evidence. Front Psychol, 13, 1041675. https://doi.org/10.3389/fpsyg.2022.1041675	Review
60	Tyrer, P., Duggan, C., Yang, M., & Tyrer, H. (2023, Jul 10). The effect of environmental change, planned and unplanned life events on the long-term outcome of common mental disorders. Soc Psychiatry Psychiatr Epidemiol. https://doi.org/10.1007/s00127-023-02520-1	Not a Social Prescribing intervention
61	Williams, M. J., & Tiedens, L. Z. (2016, Feb 2016). The subtle suspension of backlash: A meta-analysis of penalties for women's implicit and explicit dominance behavior. Psychological Bulletin, 142(2), 165-197. https://doi.org/https://doi.org/10.1037/bul0000039	Review
62	Yin, R., Huang, J., Crisp, G., & Ivers, R. (2023). Sustainable general practice. Australian Journal of General Practice, 52(5), 257-261. https://ezproxy.scu.edu.au/login?url=https://www.proquest.com/scholarly-journals/sustainable-general-practice/docview/2810210885/se-2?accountid=16926	Not a Social Prescribing intervention
63	Zisman-Ilani, Y., & Byrne, L. (2023, Apr 1). Shared Decision Making and Peer Support: New Directions for Research and Practice. Psychiatr Serv, 74(4), 427-428. https://doi.org/10.1176/appi.ps.20220407	Not a Social Prescribing intervention

Eligibility assessment

Publication: 2020, Aggar, Caruana, Thomas, Baker, NSW Social prescribing as an intervention for people with work-related injuries and psychosocial difficulties in Australia

	Inclusion Criteria	Meets Criteria Y/N	Exclusion Criteria	Meets Criteria Y/N
Publications				
	Primary research (i.e. not reviews or commentaries etc.)	Y	Review articles<br>Books, conference proceedings<br>Non-English language	Y
	Peer-reviewed journals	Y		
	English language	Y		

	Specify "social prescribing"	Y		
Studies				
Population	Any age<br>Any gender	Y	Inpatient	Y
	Living in Australian community	Y		
Intervention	Social prescription to any non-medical supports/services	Y		
Comparator	None required, no restrictions	Y		
Outcomes	No restrictions	Y		
Time	No restrictions	Y		
Study design	No restrictions	Y		

Publication: 2020, Thomas et al, Stepped-wedge cluster randomised trial of social prescribing of forest therapy for quality of life and biopsychosocial wellbeing in community-living Australian adults with mental illness: protocol

	**Inclusion Criteria**	**Meets Criteria Y/N**	**Exclusion Criteria**	**Meets Criteria Y/N**
Publications				
	Primary research (i.e. not reviews or commentaries etc.)	Y	Review articles<br>Books, conference proceedings<br>Non-English language	Y
	Peer-reviewed journals	Y		
	English language	Y		
	Specify "social prescribing"	Y		
Studies				
Population	Any age<br>Any gender	Y	Inpatient	Y

	Living in Australian community	Y		
Intervention	Social prescription to any non-medical supports/services	Y		
Comparator	None required, no restrictions	Y		
Outcomes	No restrictions	Y		
Time	No restrictions	Y		
Study design	No restrictions	Y		

Publication: 2020, Aggar, Thomas, Gordon, Bloomfield, Baker, Social prescribing for individuals living with mental illness in an Australian community setting: a pilot study

	Inclusion Criteria	Meets Criteria Y/N	Exclusion Criteria	Meets Criteria Y/N
Publications				
	Primary research (i.e. not reviews or commentaries etc.)	Y	Review articles<br>Books, conference proceedings<br>Non-English language	Y
	Peer-reviewed journals	Y		
	English language	Y		
	Specify "social prescribing"	Y		
Studies				
Population	Any age<br>Any gender	Y	Inpatient	Y
	Living in Australian community	Y		
Intervention	Social prescription to any non-medical supports/services	Y		
Comparator	None required, no restrictions	Y		

Outcomes	No restrictions	Y		
Time	No restrictions	Y		
Study design	No restrictions	Y		

Publication: 2023, Dingle et al, A controlled evaluation of social prescribing on loneliness for adults in Queensland: 8-week outcomes

	Inclusion Criteria	Meets Criteria Y/N	Exclusion Criteria	Meets Criteria Y/N
Publications				
	Primary research (i.e. not reviews or commentaries etc.)	Y	Review articles<br>Books, conference proceedings<br>Non-English language	Y
	Peer-reviewed journals	Y		
	English language	Y		
	Specify "social prescribing"	Y		
Studies				
Population	Any age<br>Any gender	Y	Inpatient	Y
	Living in Australian community	Y		
Intervention	Social prescription to any non-medical supports/services	Y		
Comparator	None required, no restrictions	Y		
Outcomes	No restrictions	Y		
Time	No restrictions	Y		
Study design	No restrictions	Y		

Publication: 2023, Jayasinghe et al, An Ounce of Prevention is Worth a Pound of Cure": Proposal for a Social Prescribing Strategy for Obesity Prevention and Improvement in Health and Well-being

	Inclusion Criteria	Meets Criteria Y/N	Exclusion Criteria	Meets Criteria Y/N
Publications				
	Primary research (i.e. not reviews or commentaries etc.)	Y	Review articles<br>Books, conference proceedings<br>Non-English language	Y
	Peer-reviewed journals	Y		
	English language	Y		
	Specify "social prescribing"	Y		
Studies				
Population	Any age<br>Any gender	Y	Inpatient	Y
	Living in Australian community	Y		
Intervention	Social prescription to any non-medical supports/services	Y		
Comparator	None required, no restrictions	Y		
Outcomes	No restrictions	Y		
Time	No restrictions	Y		
Study design	No restrictions	Y		

Publication: 2023, Ostojic et al, Codesigning a social prescribing pathway to address the social determinant of health concerns of children with cerebral palsy and their families in Australia: a protocol for a mixed-methods formative research study

	Inclusion Criteria	Meets Criteria Y/N	Exclusion Criteria	Meets Criteria Y/N
Publications				
	Primary research (i.e. not reviews or commentaries etc.)	Y	Review articles<br>Books, conference proceedings	Y

			Non-English language	
	Peer-reviewed journals	Y		
	English language	Y		
	Specify “social prescribing”	Y		
Studies				
Population	Any age<br>Any gender	Y	Inpatient	No children are patients of paediatric rehab centre
	Living in Australian community	Y		
Intervention	Social prescription to any non-medical supports/services	No intervention provided		
Comparator	None required, no restrictions	Y		
Outcomes	No restrictions	Y		
Time	No restrictions	Y		
Study design	No restrictions	Y		

Publication: 2021, Pedell et al, Combining the Digital, Social and Physical Layer to Create Age-Friendly Cities and Communities

	**Inclusion Criteria**	**Meets Criteria Y/N**	**Exclusion Criteria**	**Meets Criteria Y/N**
Publications				
	Primary research (i.e. not reviews or commentaries etc.)	Y	Review articles<br>Books, conference proceedings<br>Non-English language	Y

	Peer-reviewed journals	Y		
	English language	Y		
	Specify "social prescribing"	Y		
Studies				
Population	Any age<br>Any gender	Y	Inpatient	Y
	Living in Australian community Australian paper but does not state participants live in Australian community	Y		
Intervention	Social prescription to any non-medical supports/services	Y		
Comparator	None required, no restrictions	Y		
Outcomes	No restrictions	Y		
Time	No restrictions	Y		
Study design	No restrictions	Y		

Publication: 2019, Lawn et al, Outcomes of telephone-delivered low intensity cognitive behaviour therapy (LiCBT) to community dwelling Australians with a recent hospital admission due to depression or anxiety: MindStep™

	**Inclusion Criteria**	**Meets Criteria Y/N**	**Exclusion Criteria**	**Meets Criteria Y/N**
Publications				
	Primary research (i.e. not reviews or commentaries etc.)	Y	Review articles<br>Books, conference proceedings<br>Non-English language	Y
	Peer-reviewed journals	Y		
	English language	Y		

	Specify “social prescribing”	N- Refers to only one reference about social prescribing		
Studies				
Population	Any age<br>Any gender	Y	Inpatient	Y
	Living in Australian community	Y		
Intervention	Social prescription to any non-medical supports/services	Y		
Comparator	None required, no restrictions	Y		
Outcomes	No restrictions	Y		
Time	No restrictions	Y		
Study design	No restrictions	Y		

Publication: 2023, Woolfenden, Equity Pathways in Integrated Care in Cerebral Palsy (EPIC-CP): a pilot clinical trial of social prescribing for children and young people with cerebral palsy and their parents/caregivers

	**Inclusion Criteria**	**Meets Criteria Y/N**	**Exclusion Criteria**	**Meets Criteria Y/N**
Publications				
	Primary research (i.e. not reviews or commentaries etc.)	Y	Review articles<br>Books, conference proceedings<br>Non-English language	Y
	Peer-reviewed journals	N- RCT register		
	English language	Y		

	Specify "social prescribing"	Y		
Studies				
Population	Any age<br>Any gender	Y	Inpatient	N- children admitted to paediatric hospital
	Living in Australian community	Y		
Intervention	Social prescription to any non-medical supports/services	Y		
Comparator	None required, no restrictions	Y		
Outcomes	No restrictions	Y		
Time	No restrictions	Y		
Study design	No restrictions	Y		

Publication: 2023, Peterson, Promotion of social prescribing from within a general practice (a pilot study)

	**Inclusion Criteria**	**Meets Criteria Y/N**	**Exclusion Criteria**	**Meets Criteria Y/N**
Publications				
	Primary research (i.e. not reviews or commentaries etc.)	Y	Review articles<br>Books, conference proceedings<br>Non-English language	Y
	Peer-reviewed journals	N- RCT register		
	English language	Y		
	Specify "social prescribing"	Y		
Studies				
Population	Any age	Y	Inpatient	Y

	Any gender			
	Living in Australian community	Y		
Intervention	Social prescription to any non-medical supports/services	Y		
Comparator	None required, no restrictions	Y		
Outcomes	No restrictions	Y		
Time	No restrictions	Y		
Study design	No restrictions	Y		

Publication: 2008, Huggins et al, Socially prescribed and Self-Orientated Perfectionism as Predictors of Depressive Diagnosis in Preadolescents

	**Inclusion Criteria**	**Meets Criteria Y/N**	**Exclusion Criteria**	**Meets Criteria Y/N**
Publications				
	Primary research (i.e. not reviews or commentaries etc.)	Y	Review articles<br>Books, conference proceedings<br>Non-English language	Y
	Peer-reviewed journals	Y		
	English language	Y		
	Specify "social prescribing"	N- refers to Socially Prescribed Perfectionism		
Studies				
Population	Any age<br>Any gender	Y	Inpatient	Y
	Living in Australian community	Y		

Intervention	Social prescription to any non-medical supports/services	N- no intervention		
Comparator	None required, no restrictions	Y		
Outcomes	No restrictions	Y		
Time	No restrictions	Y		
Study design	No restrictions	Y		

Publication: 2022, Barnett et al, Transitioning to civilian life: The importance of social group engagement and identity among Australian Defence Force veterans

	**Inclusion Criteria**	**Meets Criteria Y/N**	**Exclusion Criteria**	**Meets Criteria Y/N**
Publications				
	Primary research (i.e. not reviews or commentaries etc.)	Y	Review articles<br>Books, conference proceedings<br>Non-English language	Y
	Peer-reviewed journals	Y		
	English language	Y		
	Specify “social prescribing”	N- only mentions SP for veterans is needed in the conclusion		
Studies				
Population	Any age<br>Any gender	Y	Inpatient	Y
	Living in Australian community	Y		
Intervention	Social prescription to any non-medical supports/services	N		

Comparator	None required, no restrictions	Y		
Outcomes	No restrictions	Y		
Time	No restrictions	Y		
Study design	No restrictions	Y		

Publication: 2023, Foley et al, Towards Key Principles for the Design and Implementation of Nature Prescription Programs

	Inclusion Criteria	Meets Criteria Y/N	Exclusion Criteria	Meets Criteria Y/N
Publications				
	Primary research (i.e. not reviews or commentaries etc.)	Y	Review articles<br>Books, conference proceedings<br>Non-English language	Y
	Peer-reviewed journals	Y		
	English language	Y		
	Specify “social prescribing”	N		
Studies				
Population	Any age<br>Any gender	Y	Inpatient	N- participants were health stakeholders who some work in clinical settings
	Living in Australian community	Y		
Intervention	Social prescription to any non-medical supports/services	Y		
Comparator	None required, no restrictions	Y		

Outcomes	No restrictions	Y		
Time	No restrictions	Y		
Study design	No restrictions	Y		

Publication: 2023, Dias et al, 'Safety Nets': a community based social prescribing intervention involving combined physical activity and psychoeducation for young people on mental health service waiting lists: a pilot service evaluation

	**Inclusion Criteria**	**Meets Criteria Y/N**	**Exclusion Criteria**	**Meets Criteria Y/N**
Publications				
	Primary research (i.e. not reviews or commentaries etc.)	Y	Review articles<br>Books, conference proceedings<br>Non-English language	Y
	Peer-reviewed journals	Y		
	English language	Y		
	Specify "social prescribing"	Y		
Studies				
Population	Any age<br>Any gender	Y	Inpatient	Y
	Living in Australian community	N		
Intervention	Social prescription to any non-medical supports/services	Y		
Comparator	None required, no restrictions	Y		
Outcomes	No restrictions	Y		
Time	No restrictions	Y		
Study design	No restrictions	Y		

Data extraction

Study	**Aim**	**Design Setting Participants**	**Social Prescribing Strategy**	**Intervention Comparator**	**Outcome domain/s**	**Outcome measure/s**	**Findings**
Aggar, Caruana<br><br>Social prescribing as an intervention for people with work-related injuries and psychosocial difficulties in Australia	To describe the economic, social, health service utilisation, and quality of life outcomes of injured workers.	Mixed methods i.e., questionnaires and interviews.<br><br>Sydney, Australia.<br><br>*n* = 175, 18–65 years.<br><br>Unable to return to work after a work-related injury acquired 6 months – 3 years prior or returned to work on reduced hours/duties.	GP identifies as experiencing psychosocial difficulties and is likely to benefit from increased social participation.<br><br>Referred to care coordination service for holistic needs assessment, care planning, linkage and referral with follow-up contact.	12-week program. Follow-up post-intervention).<br><br>Activities included arts and crafts, yoga and relaxation, equine therapy, and social groups.<br><br>Additional referrals to financial and housing support, relationship counselling, and mental health support groups.	Global Well-Being and Needs	Camberwell Assessment of Needs Short Appraisal Schedule (CANSAS).<br>WHO Quality of Life Brief Assessment (WHOQoL-Brief).	Social prescribing is effective in improving the overall well-being of injured workers with psychological difficulties.<br><br>Benefits included increased social connectedness, confidence and ability to return to work, and reduced pain, distress, and health service needs.
					Social Well-Being.	The UCLA 3-item Loneliness Scale (UCLA-3).<br>Number of people participants could count on.<br>Satisfaction with Social Support.	
					Emotional Well-Being.	10-item Kessler Psychological Distress Scale (K10).	
					Physical Well-Being.	Pain Rating Scale (1 item, rated 0 – 10).<br>EQ-5D-5L Health Thermometer (EQ5D).	
					Patient/Service User Experience	Program satisfaction ratings.<br>Interview data.	
					Social Determinants of Health	Confidence in returning to work.	
					Health Service Utilisation	Frequency of hospitalisations and other health services.<br>Interview data.	
					Occupational/ Economic Participation.	Capacity for work.<br>Current employment.<br>Interview data.	
					Feasibility	Frequency of current volunteering.	
Aggar, Thomas	Improve quality of life, and social and economic participation of people with diagnosed mental illness.	Exploratory, quantitative, longitudinal design.<br><br>Sydney, Australia.<br>*N* = 13, 18–65 years.	GP identifies unmet biopsychosocial needs and enrols in the program.<br><br>Link workers conduct holistic	10-week program. Follow-up six months post-baseline.<br><br>All participants attend weekly arts and crafts	Global Well-Being and Needs	Global Quality of Life—WHOQoL-Brief.<br>Camberwell Assessment of Needs Short Appraisal Schedule (CANSAS).	Significant improvement in physical and psychological QoL, health satisfaction and self-perceived health status.

		Living in the community in the Sydney Local Health District.<br><br>Diagnosed with serious mental illness likely to last 6 months or longer.	needs assessment, care planning, linkage, and referral with follow-up contact.	groups (2–3 hours).<br><br>Additional referrals to chronic disease management, acute care ‘hospital in the home’, financial and housing support, relationship counselling, and mentoring programs.	Social Well-Being.	UCLA 3-item Loneliness Scale.	No significant differences in social participation self-rated loneliness, and economic participation.
					Emotional Well-Being.	The Kessler Psychological Distress Scale (K10).	
					Physical Well-Being	EuroQol Health Thermometer EQ5D.	
					Economic Return	Participation in paid employment (yes/no) in the previous 2 weeks.	
Social prescribing for individuals living with mental illness in an Australian community setting: a pilot study	Improve loneliness and wellbeing and decrease health service usage among people experiencing loneliness or social isolation.	Non-randomised control trial.<br><br>QLD, Australia<br>*n* = 114, >=18 years.<br><br>Experiencing loneliness or social isolation based on self-report and/or identified by their health or social care workers.<br><br>Frequent GP attenders (>= 12 visits per year for 2 years).	Referrals from GPs and hospitals (*n* = 20), community services (*n* = 20), and self/family referrals (*n* = 13).<br><br>Link worker refers to community group activities.	8-week community group program, including art and creative activity, physical and outdoor activity, educational courses, and others).<br><br>GP Treatment as usual (TAU).<br><br>Post intervention follow up (8-weeks).	Social Well-Being.	8-item UCLA Loneliness Scale (ULS-8).<br>Social anxiety: 3-item Social Phobia Inventory (mini-SPIN).<br>Social trust: adapted version of the Cognitive Trust in Service Relationships Scale.	Improvements in loneliness, social trust, wellbeing were significantly different for social prescribing group.<br><br>Psychological distress and social anxiety were not significantly different for social prescribing group but had a small to medium effect size.
					Emotional Well-Being.	6-item Kessler Psychological Distress Scale (K6).<br>Warwick Edinburgh Mental Wellbeing Scale.	
					Health Service Utilisation	Frequency of hospital visits.<br>Attendance at GPs, allied health (counsellor, psychologist, psychiatrist, social worker), and community mental health services.	
					Economic Return	Frequency of work (past month).	
					Feasibility	Percentage of participants retained at the 8-week assessment period.	
Dingle, Sharman<br>A controlled evaluation of social prescribing on	To improve the quality of life and biopsychosocial wellbeing of community-	Stepped-wedge cluster randomised design.<br><br>Sydney-Gold Coast, Australia.	GP referral to care coordination service (PCCS) where link workers	Initial 10-week control period.<br>Follow-up post-intervention and 5 weeks	Global Well-Being and Needs	Global Quality of Life—WHOQoL-Brief.	N/A
					Social Well-Being	UCLA 3-item Loneliness Scale.	

loneliness for adults in Queensland: 8-week outcomes	living adults with diagnosed severe mental illness.	*n* = 140 (planned), >=18 years.<br><br>Diagnosed with severe and persistent/complex mental illness (mood or psychotic disorder).	complete a holistic needs assessment and enrol participants.<br><br>Participants also receive usual care including referral to other health and welfare services.	post-intervention.<br><br>10 weekly 90-minute forest therapy sessions in groups of 6–10.		Work and Social Adjustment Scale.	
					Emotional Well-Being.	Depression—Patient Health Questionnaire-9.<br>Anxiety—Generalised Anxiety Disorder Questionnaire.	
					Physical well-being	The Health Confidence Score.<br>Physical Health Subscale of WHO-QoL-Brief.	
					Social Determinants of Health	Work and Social Adjustment Scale.	
					Health Service Utilisation	Frequency of ambulance use, hospital visits and admissions.<br>Nights spent in hospital.<br>GP visits.<br>Allied health, and community health service utilisation.	
Study Protocols Thomas, Baker<br><br>Stepped-wedge cluster randomised trial of social prescribing of forest therapy for quality of life and biopsychosocial wellbeing in community-living Australian adults with mental illness: protocol	Develop sustainable community program to prevent obesity and related lifestyle diseases and enhance wellbeing among community dwelling residents of Circular Head, TAS.	Prospective multi method design i.e., questionnaires, focus groups, health system data, allied health data.<br>Australia.<br><br>Circular Head, TAS.<br><br>Convenience sample.	Recruitment from community-level lifestyle screening at local events (e.g. sporting events) and workplaces, GPs and allied health, and trainee health professionals (e.g. nutrition and exercise science).<br><br>Link worker assessment and referral including coproducing health goals and action plans.	3-year pilot phase.<br>No specified follow-up timeline.<br><br>Peer education, health screening, service access, and workforce connectivity.<br><br>Improve food literacy, physical literacy and activity levels, mental health, community connectedness, and reduction of social isolation.	Global Well-Being and Needs	Subjective Well-Being - not specified.<br>Quality of Life - not specified.	Circular Head residents will co-design a sustainable solution to health and wellbeing challenges and increased access to peer and other support workers.
					Social Well-Being.	Development of Social Networks (not specified).<br>Social return on investment (not specified).	
					Physical Well-Being.	Physiological changes (not specified).<br>Medication usage (not specified).	
					Patient/Service User Experience	Behaviour Change - not specified.	
					Health Service Utilisation.	Frequency of Health Service Access.	
	To assess the social prescribing program	Randomised control trial.	Participants in the social prescribing group will	Intervention duration unknown.	Social Well-Being	Unmet Social Needs - adapted WECARE tool.	N/A.

	implementation and its health outcomes among parents of cerebral palsy patients.	NSW, ACT, Australia.<br><br>*n* = 120, parents/caregiver of a child (0-18 years) with cerebral palsy, who is a patient of one of six predetermined tertiary Paediatric Rehabilitation Departments.<br><br>Report at least one unmet social need from the following: Childcare or schooling; Government benefits and vouchers; Housing; Food; Bills; Transport.	receive a resource pack and be allocated a link worker.	Follow up at 3 months and 6 months post-randomisation.<br><br>Link workers will consider family needs to refer a case-by-case intervention.<br><br>Comparator unknown.	Emotional Well-Being	K-6 Distress Scale.	
					Patient/Service User Experiences.	Participants experiences – not specified.<br>Barriers and enablers to social prescribing.<br>PROMIS Scale.<br>PROMIS Parent Proxy Scale<br>PROMIS Paediatric Scale for children/young people >8 years who can self-report.	
					Feasibility	Recruitment rates.<br>Uptake of intervention.<br>Follow-up of participants.	
					Fidelity	Type of social prescribing activities referrals, inquires and attendance.	

MMAT/quality appraisal

	SCREENING QUESTIONS		1. QUALITATIVE STUDIES					2. RANDOMIZED CONTROLLED TRIALS					3. NON-RANDOMIZED STUDIES					4. QUANTITATIVE DESCRIPTIVE STUDIES					5. MIXED METHODS STUDIES				
First author	S1. Are there clear research questions?	S2. Do the collected data allow to address the research questions?	1.1. Is the qualitative approach appropriate to answer the research question?	1.2. Are the qualitative data collection methods adequate to address the research question?	1.3. Are the findings adequately derived from the data?	1.4. Is the interpretation of results sufficiently substantiated by data?	1.5. Is there coherence between qualitative data sources, collection, analysis and interpretation?	2.1. Is randomization appropriately performed?	2.2. Are the groups comparable at baseline?	2.3. Are there complete outcome data?	2.4. Are outcome assessors blinded to the intervention provided?	2.5 Did the participants adhere to the assigned intervention?	3.1. Are the participants representative of the target population?	3.2. Are measurements appropriate regarding both the outcome and intervention (or exposure)?	3.3. Are there complete outcome data?	3.4. Are the confounders accounted for in the design and analysis?	3.5. During the study period, is the intervention administered (or exposure occurred) as intended?	4.1. Is the sampling strategy relevant to address the research question?	4.2. Is the sample representative of the target population?	4.3. Are the measurements appropriate?	4.4. Is the risk of nonresponse bias low?	4.5. Is the statistical analysis appropriate to answer the research question?	5.1. Is there an adequate rationale for using a mixed methods design to address the research question?	5.2. Are the different components of the study effectively integrated to answer the research question?	5.3. Are the outputs of the integration of qualitative and quantitative components adequately interpreted?	5.4. Are divergences and inconsistencies between quantitative and qualitative results adequately addressed?	5.5. Do the different components of the study adhere to the quality criteria of each tradition of the methods involved?
Aggar 2020	Yes	Yes																					Can't tell	Yes	Yes	Yes	Yes
Thomas 2020	Yes	Yes						Yes	Yes		No																
Aggar 2021	Yes	Yes											Can't tell	Yes	Can't tell	No	Yes										
Dingle 2022	Yes	Yes											No	Yes	Yes	No	Can't tell										
Jayasinghe 2023	Yes	Yes																					Yes	Yes	Can't tell	Can't tell	Can't tell
Pedell 2021	Yes	Yes	Yes	Yes	Yes	Yes	Yes																				
Woolfenden 2022	Yes	Yes						Yes	Yes	Can't tell	Can't tell	Can't tell															
Peterson 2022	Yes	Yes											No	Yes	Can't tell	No	Can't tell										
